# IPLOT‐VKA: An Integral‐Method Powell‐pLOT‐Enhanced Visual Kinetic Analysis for the Determination of Orders of Reaction

**DOI:** 10.1002/chem.202401914

**Published:** 2024-12-13

**Authors:** Alessandro Landi, Guglielmo Monaco

**Affiliations:** ^1^ Department of Chemistry and Biology “A. Zambelli” University of Salerno Via G. Paolo II, 132 Fisciano, SA 84084 Italy; ^2^ Department of Chemistry and Biology “A. Zambelli” University of Salerno Via G. Paolo II, 132 Fisciano, SA 84084 Italy

**Keywords:** Order of reaction, Kinetic constant, Elasticity coefficient, Master curve, Visual kinetic analysis

## Abstract

Determination of partial orders of reactions in kinetics is a general entry step for any mechanistic investigation; recently, considerable attention has been given to the benefits of using visual methods to help in this task, such as easy utilization and data‐efficiency. In this respect, we here revisit and improve the classic method by Powell and Margerison for kinetic analysis. For the first time, analytical equations for a bimolecular reaction have been worked out for all values of partial orders of reactions, and the solutions have been implemented in a free, open‐access and easy‐to‐use Web‐application, named IPLOT‐VKA, which also allows estimation of the errors on the kinetic constant, and residual standard error on concentrations. Several examples, taken from reference teaching experiences and from recent literature in catalysis show the efficacy and accuracy of our approach, which also has the advantage of requiring less experimental runs than other visual methods currently available.

## Graphical abstract

Methods for the determination of partial orders of reaction have been surveyed and expanded. A free online application named IPLOT‐VKA, after Integral‐method Powell‐pLOT‐enhanced‐Visual Kinetic Analysis, has been developed to speed up the determination of partial orders of reaction in chemical kinetics. Applications to classical teaching experiments and current literature in catalysis are reported.

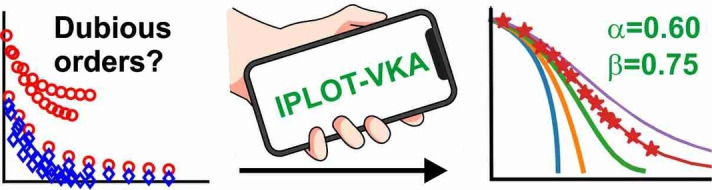

## Introduction

The determination of partial orders in reactants and catalysts is widely used in both academia and industry. Several papers, in relatively recent literature, have addressed this fundamental and basic task, proposing novel techniques collectively known as visual kinetic analysis (VKA).[^1^, ^2^, ^3^, ^4^, ^5^] These techniques have generated a lot of interest, because they are easy to use and require less data than standard textbooks’ methods to provide useful information. Actually, much of their merits are shared by older approaches, like that of Powell and its thorough extension by Margerison,[^6^, ^7^, ^8^, ^9^, ^10^] which were however presented in a nowadays out‐fashioned protocol making use of translucent graph‐paper.^[9]^ Possibly, the fact that the first presentation of these two methods is found in books rather than journal papers has made them less known than other approaches, to the point that they are never cited in the VKA literature.[^1^, ^2^, ^3^, ^4^, ^5^]

To fill this gap and provide the research community with a fast and ready‐to‐use Web Application for kinetic analysis, the analytical tools developed by Margerison have been further extended and given a modern digital form. Concentrations of reactants for a reaction with two reactants following a kinetics with a simple rate law have been worked out analytically for all values of partial orders of reaction, and the initial burden of creating the master curves needed for the analysis, as well as the time‐consuming pre‐processing of data in case of multiple kinetic runs, have been rendered unnecessary thanks to the development of a fast, open‐access application, developed in python, an interpreted language which is gaining momentum in Chemistry.^[11]^ The application, named IPLOT‐VKA (after Integral‐method Powell‐pLOT‐enhanced‐Visual Kinetic Analysis) comes with an easy web interface (http://alelandi.pythonanywhere.com/), thus offering the possibility to considerably ease the initial investigation of kinetic data. Our method also comes with quantitative uncertainties on the kinetic constants and residual standard errors on concentrations, something lacking in previous VKA methods, but necessary to assess the validity of the results.^[5]^

### Results and Discussion

In the following we will give a basic discussion of our method; further mathematical details can be found in the Supplementary Information. We will be interested in a reaction

(1) aA+bB→pP+qQ,

where a , *b*, *p*, *q* are the stoichiometric coefficients. Before starting, we remark that, surprising as it might be, notation in kinetics is not engraved in marble; limiting to the partial order of reaction of reactant A, there are four different suggestions in IUPAC recommendations:[^12^, ^13^] *m_A_*, *n_A_*, *a* and *α*. We will here adopt the last one, which matches with the majority of recent textbooks.

The rate law for reaction (1) generally has a complicated mathematical expression, as a consequence of the many steps required to obtain products. For what Margerison called a *simple* rate equation, the rate of disappearance of A, when all other parameters (temperature, pressure, catalyst concentration, solvent…) are kept fixed, can be written as

(2) $$R_{A}=-\frac{d\left[A\right]}{dt}=k_{A}{\left[A\right]}^{\alpha }{\left[B\right]}^{\beta }$$

with constant values of $k_{A}$ , *α* and *β* (the last two being the partial orders of reaction). A simple rate equation occurs for elementary (single‐step) reactions (with α=a and β=b ), but it can also occur for complex (multistep) reactions.

Identifying the rate equation as *simple* is made possible by the precision and, to a large extent, the diversity of the data, and, upon widening the dataset, simple rate laws can fall into the complicated rate laws domain. Nevertheless, for any rate law, simple or complicated, focusing on sufficiently narrow concentration ranges, Equation (2) still holds, and so it is possible to define the orders as $\alpha {=\left(\frac{\partial lnR_{A}}{\partial ln\left[A\right]}\right)}_{\left[B\right]}$ and $\beta {=\left(\frac{\partial lnR_{A}}{\partial ln\left[B\right]}\right)}_{\left[A\right]}.$ [^10^, ^13^] Notably, this general definition is not adopted by older IUPAC recommendations^[14]^ and by the majority of textbooks, which require the rate to strictly follow Equation (2), in all the data domain, to properly define orders of reaction. Quite recently, orders of reaction changing with concentration have been referred to as “elasticity coefficients”.[^15^, ^16^] Named as they will, orders of reaction (and their “elastic” change with concentration) are that piece of information which comes useful to draw up mechanistic models from kinetic data. It should be kept in mind that, while partial orders of reaction which are neither integers nor half‐integers could bring some hints in modelling the mechanism of a complex reaction, they could also stem from overfitting. Therefore, their adoption as sound parameters of a simple kinetic law should require a statistically significant improvement of the goodness of fit. This point is discussed into detail in Section S5 of Supplementary Information, and we will see its application for the examples analyzed below.

Let us first focus on the simplified case A→ P, with rate law

(3) $$R=\frac{d\left[P\right]}{dt}=-\frac{d\left[A\right]}{dt}=k{\left[A\right]}^{\alpha }.\;$$

Rather than trivial, or a mathematical curiosity when β *=*0, this is an important case, found e. g. when working with a starting concentration of B so much larger than that of A, that it can be considered constant along the kinetic run (isolation or flooding method conditions, when the effective kinetic constant is $k^{'}≅k{\left[B\right]}_{0}^{\beta }$ ); this approach is widely used,^[10]^ since it allows simplifying the rate expression.

Experimentally, one rarely determines rates, but rather concentrations and times, so that, in order to have quantities to be compared, a preliminary step in data analysis requires either the estimation of the rates from experimental concentrations (method of initial velocities) or the determination of model concentrations through the integration of Equation (3). The latter operation, in turn, can be performed either numerically (as in Variable Time Normalization Analysis, VTNA,^[4]^ see Supplementary Information) or analytically, as in Powell‐Margerison plots.^[6]^ In this respect, it must be remarked that, as compared to the method of initial velocities, the integral method has the merit of using all the experimental data, and can be applied even to a single kinetic run.

For the integral method, preliminary separation of variables in Equation (3) followed by a multiplication to have adimensional quantities on left and right hand side of Equation (3) gives

(4) $$\;\text{-}{\left[A\right]}_{0}^{\alpha -1}\frac{d\left[A\right]}{{\left[A\right]}^{\alpha }}=-\frac{d\varphi }{\varphi^{\alpha }}\;\;\;\;\;\;\;\;\;\;\;\;\;\;\;\;\;\;=\;k{\left[A\right]}_{0}^{\alpha -1}dt\;\;\;\;\;\;\;\;\;$$

where we have introduced the fraction

$\varphi =\left[A\right]/{\left[A\right]}_{0}\;$ of unreacted substrate A. Integration yields then

(5) $$\;\int_{\varphi }^{1}\frac{d\varphi }{\varphi^{\alpha }}=\Delta f\left(\varphi ;\alpha \right)\;\;\;\;\;\;\;\;\;\;\;\;\;\;=\;k{\left[A\right]}_{0}^{\alpha -1}t=\;k\tau =\theta \;\;\;\;\;\;\;\;\;\;$$

where $\Delta f=f\left(1;\alpha \right)-f\left(\varphi ;\alpha \right)$ is a function of *φ*, depending parametrically on the order *α*, τ is a concentration‐weighted time, and θ is Margerison's dimensionless reduced time. This reduced time θ coincides with the fractionation time (the time needed to reduce the initial concentration ${\left[A\right]}_{0}$ to $\left[A\right]=\varphi {\left[A\right]}_{0}$ ) when $k{\left[A\right]}_{0}^{\alpha -1}t^{*}=1\;$ , with t* the unit of time, e. g. 1 s. Δf is a monotonous function of φ , so that either Δf or φ can be equivalently chosen as the variable to follow the kinetics. We shall see later the utility of introducing these quantities φ and θ.

It should be noted that, in terms of φ (or Δf ) and θ (or τ ) variables, the kinetic plot is independent of the initial concentration ${\left[A\right]}_{0}$ for the correct order of reaction α . Therefore, if two kinetic runs obtained with different initial concentration overlap only at shorter values of the concentration‐weighted time τ , there is evidence of a change of mechanism, which, in the simplest cases, can be product inhibition or catalyst deactivation.^[2]^

The experimental determination of the kinetic constant, for a given order α , requires at least a pair of measures of concentration and time (hence $\Delta f=f\left(\varphi_{1};\alpha \right)-f\left(\varphi_{2};\alpha \right)$ , and ${\tau =\tau }_{2}-\tau_{1}$ ). Any pair should give the same constant, so that a criterion to select the correct order is the constancy of the kinetic constant obtained for different pairs.^[17]^ The choice of the pairs to be used for this task has been a matter of discussion.[^6^, ^18^] The choice of the *m* consecutive pairs ($\tau_{i-1},\tau_{i}$ ) turns out giving too much weight to the first and last point of the run, while the choice of the *m* pairs ($\tau_{0},\tau_{i}$ ) has been criticized because it gives excessive weight to the first point.[^6^, ^18^] Interestingly, least‐squares refinements, Δf vs τ , can be shown to coincide with a weighted average over all $\frac{m\left(m+1\right)}{2}$ pairs,[^19^, ^20^] and least‐squares with an intercept have been judged as advantageous in case of difficulty in identifying the start of the reaction.^[6]^ However, the intercept should be introduced only for the best kinetic model, in order to avoid getting unphysical time delays stemming from the limitations of the model.

We remark that, in absence of experimental errors, for a dataset following a simple rate law, all these estimates should lead to the same order of reaction. As they can be easily obtained, we think it useful to compute all of them, inasmuch as significant discrepancy between these estimates could be a suggestion to select better data or abandon least squares in favor of robust statistics.[^19^, ^20^] The present Web‐App version of IPLOT‐VKA considers two of them to select the best model: the first‐point‐*i*‐th‐point pairs (corresponding to a translation of a single point in Powell plot, see below), and least‐squares without intercept. When the best order is found, averages of kinetic constants are determined with statistical weights out of three linear fits: first‐point‐*i*‐th‐point pairs, least squares with intercept and least squares without intercept.

Although model selection through the smallest variation of the kinetic constant can be performed entirely in a blind numerical fashion, the confidence of the results can take advantage of visualization techniques. To this end, we remark the usefulness of the quantities introduced in Equation (5), inasmuch as plots of $\Delta f\left(\varphi ;\alpha \right)$ against τ turn out linear only for the correct order of reaction α . Useful as it might be, this visual corroboration, intrinsic with the integral method, can be of limited help, if similar linear plots are obtained for different orders, which happens in particular for reactions followed up to low conversions. A further discrimination among orders can be obtained by Powell's method: the plot of the fraction of unreacted substrate φ vs the decimal logarithm of time, and the comparison with master curves, which (i) can be obtained setting $k{\left[A\right]}_{0}^{\alpha -1}t^{*}=1$ , and (ii) have a peculiar shape for any order of reaction. Search for best superposition of experimental data and master curves, Δf vs ${log}_{10}\theta$ , which in the past were carried out with the help of translucent graph paper, allows determination of the best order of reaction. Moreover, as the reduced time θ is independent of both the kinetic constant and the initial concentration, the shift among the logarithm of the experimental times and the master curves (corresponding to logarithms of reduced times) carries information on the kinetic constant.

**Figure 1:**
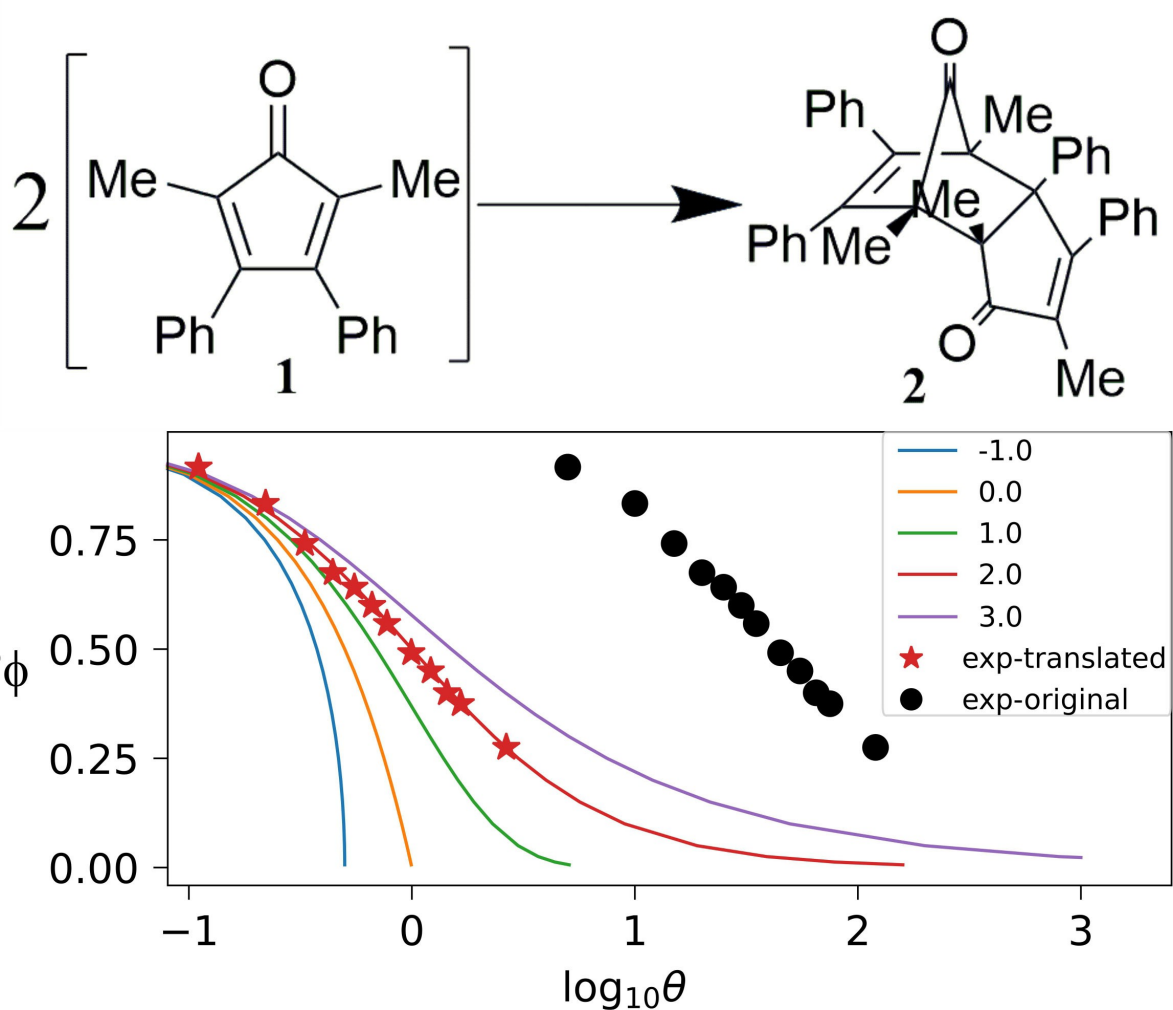
The original Powell plot method applied to the dimerization of **1**.^[21]^ By translation of the experimental entries (black dots: $\varphi =\left[1\right]/{\left[1\right]}_{0}$ vs $\log_{10}\frac{t}{t^{*}}$ ), the only good matching is obtained for Powell master curve with α =2. More superpositions with different orders are given in the Supplementary Information. The amount of translation on the *x*‐axis, $\log_{10}\frac{t}{t^{*}}-\log_{10}\theta =-\log_{10}k{\left[1\right]}_{0}^{\alpha -1}t^{*}=1.66,$ together with ${\left[1\right]}_{0}=5.3\;10^{-3}\;mol/L$ , t* =1min , gives *k=*4.17±0.02 L mol^−1^ min^−1^.

As an example, the classic teaching experiment of the second‐order dimerization of 2,5‐dimethyl‐3,4‐diphenylcyclopentadienone^[21]^ **1** is shown in Figure 1.

Incidentally, the same paradigmatic simple second‐order dimerization of **1** (Figure 1) has been recently adopted for another teaching experiment on thermodynamics,^[22]^ implying the need of the reverse process: the mechanism is complex, and the orders change upon changing concentration, so that, for purists of the older IUPAC recommendation,^[14]^ even this teaching‐level second order kinetics should require elasticity coefficients, as discussed above.

**Figure 2:**
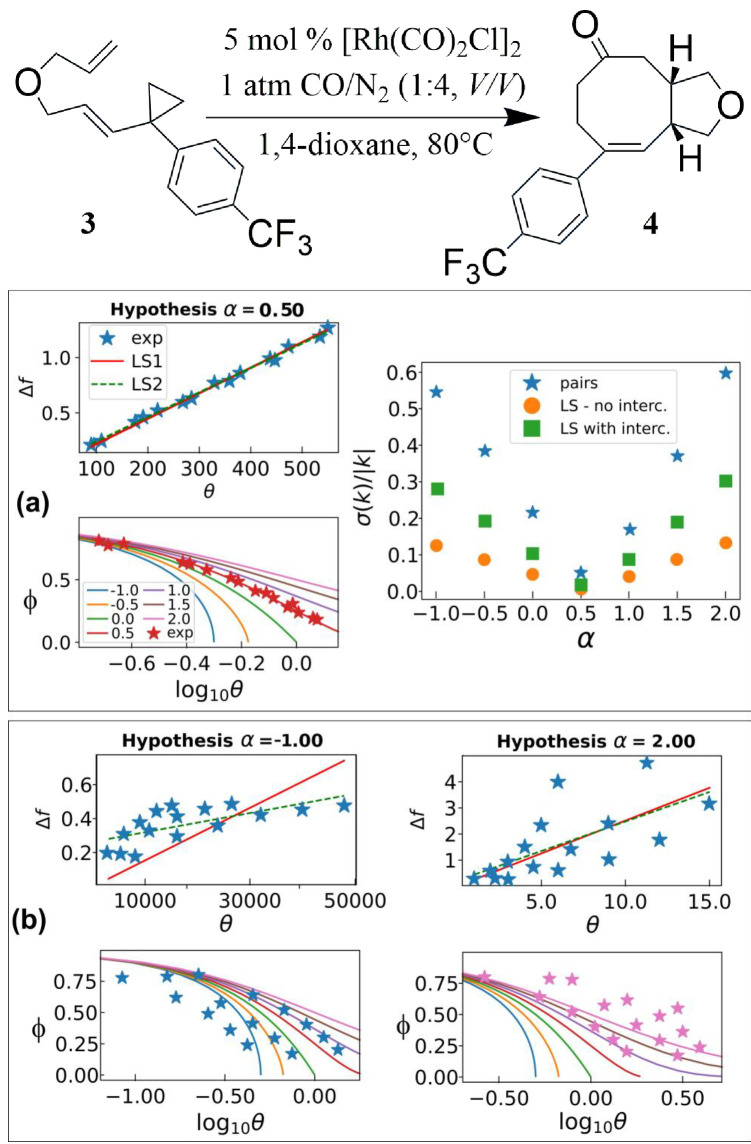
The IPLOT‐VKA method applied to the kinetic data for the cycloaddition described in ref. [23]. Scheme of the reaction (top) and kinetic analysis. In Powell‐Margerison plots, the data have been translated according to the kinetic constants determined assuming order of reaction α=0.5 (panel a), −1 (panel b, left) and 2 (panel b, right). In the middle‐right panel, the relative error obtained with different averaging methods for the competing orders is shown (LS stands for least squares).

Powell's original idea considered a single kinetic run. If more runs are considered at once, time data must be pre‐multiplied by different factors ${\left[A\right]}_{0}^{\alpha -1}$ for each tested order α , thus rendering the classic translucent graph paper approach impractical. Nevertheless, this does not prevent digital applications, as the Web Application presented here, whose working features can also be found in the Supplementary Information. As an example of the use of Powell‐Margerison plots to multiple kinetic runs, we show the determination of partial order in substrate for the rhodium‐catalyzed [5+2+1] cycloaddition of ene−vinylcyclopropanes and carbon monoxide, recently determined to be 0.5. This piece of information has been used to propose a pre‐equilibrium between a resting state dimeric form of the catalyst and an uncoordinated substrate **3** as reagents, and 2 molecules of monomeric active catalyst as products.^[23]^ This is a bimolecular reaction, but the second reactant, CO, is held constant by bubbling, so that data analysis coincides with that of a monomolecular reaction. The same partial order is recovered by IPLOT‐VKA (Figure 2a). It can be seen that for competitive models with close order of reaction, the presence of the three kinetic runs can be guessed from the unfit of the data out of the master curve (Figure 2b). Refinement of the order on a finer 0.1‐step grid (leading to α=0.6) can be judged as overfitting (see Supplementary Information).

A further improvement by Margerison to Powell's method considers the A+B reaction, Equation (1), directly: without the large excess condition for one of the reactants. The study of its kinetics requires a considerable increase of the number of mathematical formulae for the many different combinations of partial orders (see Supplementary Information, in particular Equation (S11–S13)), yielding to integrals $\Delta f\left(\varphi ;\alpha ,\beta ,c\right)$ , which depend parametrically on the two partial orders and on Margerison's parameter $c=\frac{{\left[B\right]}_{0}}{{\left[A\right]}_{0}}\frac{a}{b}-1,$ which quantifies the deviation from the stoichiometric condition. We find that the general integral, can be expressed in terms of the hypergeometric function _2_ *F*_1_,^[24]^ a result that, to the best of our knowledge, has never been reported before. With no recourse to this special function, Margerison published 15 analytical integrals for integer and half‐integer values of reaction orders. We have extended the list for α and β to integers and half‐integers in the range [−1,+3], obtaining 79 analytical integrals, which are reported in the Supplementary Information. Together with the hypergeometric function results, they allow to cover analytically all the domain for the partial reaction orders.

**Figure 3:**
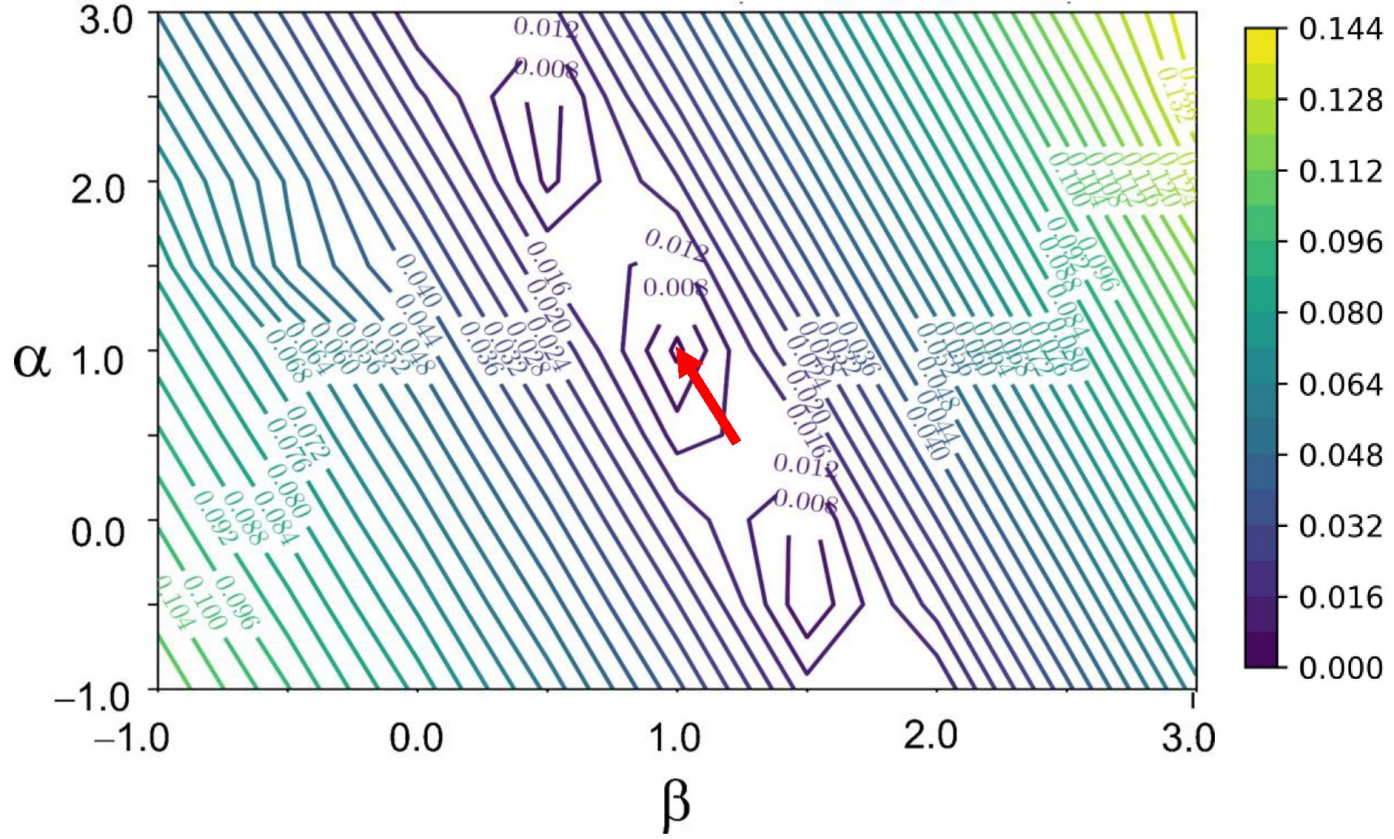
Contours of relative errors on the kinetic constant as a function of the two partial orders α and β , using the kinetic data for the S_N_2 reaction reported in ref. [17]. The correct partial orders of reactions (α=β=1 , highlighted by the red arrow) are exactly identified as the points with the lowest errors, as can be appreciated from the contour curves. In addition to the global minimum, local minima can also be seen.

In general, for the A+B reaction, Powell‐Margerison plots (or any numerical integration like those obtained with VTNA) should be tried for several values of the pair (α ,β ). The analytical equations allow performing these trials straightforwardly (this “systematic search” is also implemented in our Web‐Application). In Figure 3 we report the error on kinetic constant computed for different values of partial orders for the classic S_N_2 reaction discussed in Benson's reference textbook: in this case the partial orders are correctly identified (α=β=1 ) on a 0.5 units grid. Deviation from these values on a finer 0.1‐step grid can be safely considered as overfitting (see Supplementary Information). To the best of our knowledge, this is the first time that visual methods have been used over a two‐reagents reaction with a single kinetic dataset.

While this systematic search in the two‐dimensional (α ,β ) space offers insight in the kinetics, enabling to figure out possible competing simple rate laws, simplified approaches have also been proposed. In the VTNA method, for each partial order (e. g. α in reactant A), at least two different runs are realized keeping fixed the initial concentration of the other reactant (e. g. B), and the product formed is compared with a time weighted by a power of the concentration of the investigated reactant (A). The power leading to the best match of the different runs is the partial order of reaction α .^[4]^ It can be realized that, according to this approach, at least 3 runs are required. Alternatively, as in stoichiometric conditions the kinetics simplifies to that of an unimolecular reaction with total order *n*= α +β , the total order can be first determined from a stoichiometric mixture of reactants (a situation identified by a zero value of the Margerison's parameter c ), and then the search for one of the partial orders requires a one‐dimensional scan of possibilities, considering a non‐stoichiometric mixture. Using the latter method, here introduced and implemented in our Web‐Application, the minimum number of trials reduces to 2, instead of the 3 of VTNA.

**Figure 4:**
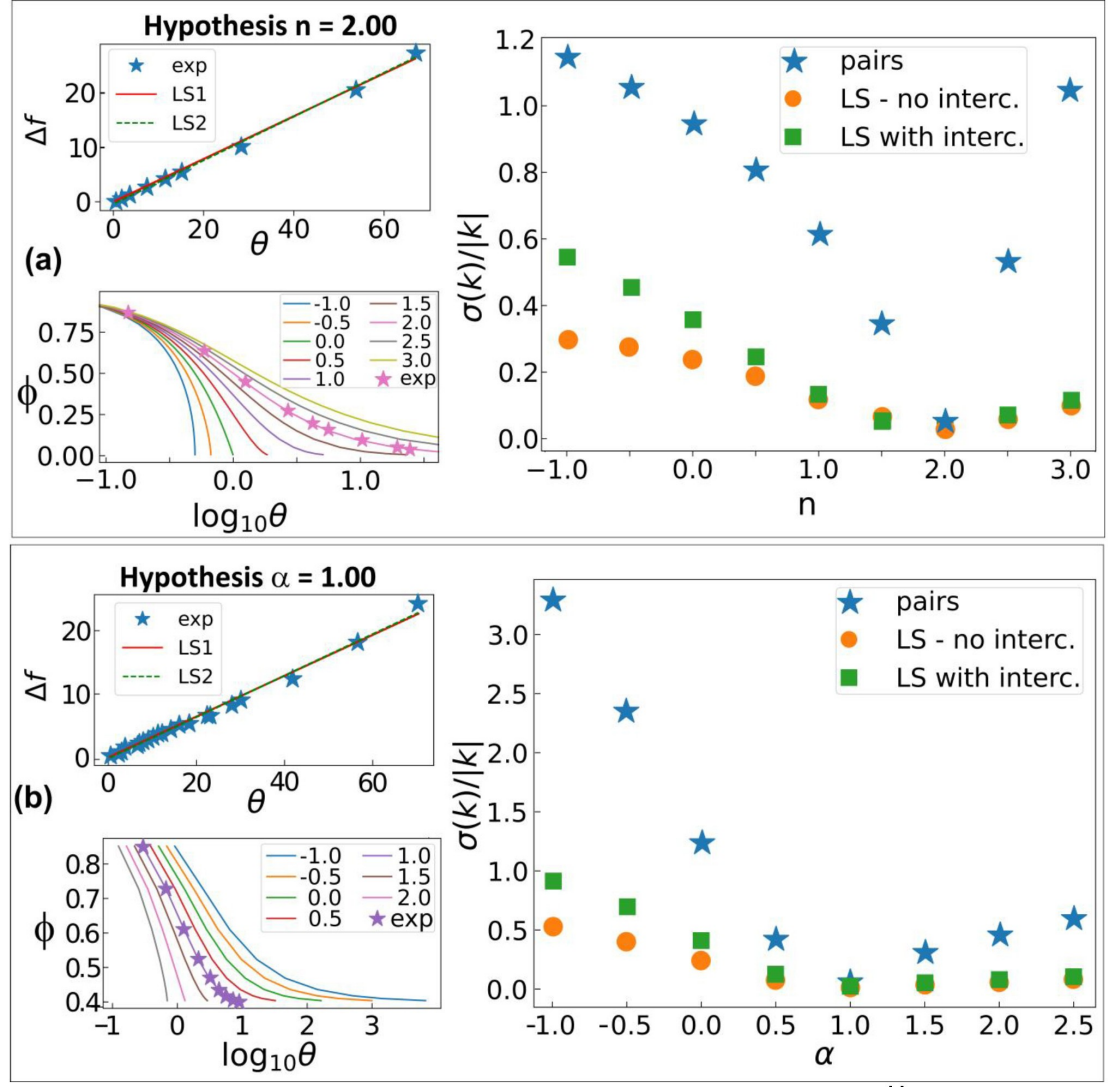
The IPLOT‐VKA method applied to the kinetic data reported in Figure 2 of ref. [4] Plots for the best total (partial) order corresponding to the integration method or the Powell‐Margerison plots are reported in the two top (bottom) panels on the left. On the right, relative errors on the kinetic constant are shown also for trials not reported in figure (LS stands for least squares). The kinetic constant is determined as *k’*=(39.5±0.06) ⋅ 10^−2^ M^−1^ h^−1^ (considering that the two runs have the same concentration of catalyst, 0.01 M, and that the order of catalyst has been set to 1, the value of *k=k’*/(0.01 M)^1^ reasonably compares with that reported in ref. [4]: *k=*34.7 M^−2^ h^−1^).

As an example of this route for the determination of kinetic parameters we used only 2 runs (one in stoichiometric conditions) out of the 3 runs used in Figure 2 of ref. [4] to exemplify the VTNA method. Total and partial orders *n*, α and β are determined as 2, 1 and 1, in agreement with the VTNA method (see Figure 4).

A further example, built on the 3 datasets generated in ref. [4] using the kinetic parameters experimentally determined in ref. [25], can be found in the Supplementary Information (Figure S4). In that case, the computation of residual standard errors on concentrations allows appreciating that a finer grid in step of 0.1 for the orders of reaction, yielding non‐half‐integer orders (α =0.6, β=0.7 ), is statistically significant. We obtain the same result using only two datasets (one in stoichiometric conditions, see Supporting Information for details).

The non‐half‐integer orders suggest the occurrence of a complicated rate law. Indeed, the mechanism of reaction proposed and successfully tested in ref. [25] stems from a double Michaelis‐Menten system, for which the rate has the concentrations of the reagents (water and epoxide) both at the first power in the numerator and as a second order polynomial in the denominator, implying that no pair of α and β orders can be straightforwardly identified, and any choice, even if statistically sound, will likely lose its optimality upon widening the dataset.

We remark that the method presented here can be straightforwardly applied to a vast number of reactions with two reactants with or without catalyst, as the logarithm of the ratio of the constants obtained with different concentration of catalyst gives access to the order in catalyst. Consideration of more than two reactants should instead require going back to the large‐excess condition or to the VTNA method.

## Conclusions

In conclusion, we have presented a significant expansion of the kinetic analysis methods pioneered by Powell and Margerison, offering a comprehensive approach to determine the orders of reaction and the kinetic constant. We have derived analytical expressions to study A+B reactions following a simple rate law, covering, for the first time, all possible values of partial orders in the range [−1, +3]. This advancement enables researchers to systematically explore the kinetics of such reactions, facilitating a deeper understanding of complex chemical processes.

Furthermore, the development of our user‐friendly online application, IPLOT‐VKA, accessible at the link http://alelandi.pythonanywhere.com/, represents a significant leap forward in the realm of Visual Kinetic Analysis. Our freely accessible tool not only allows for the estimation of kinetic constants and errors but also empowers researchers to perform a systematic trial search for the best partial orders in A+B reactions. By offering analytical calculations of partial orders, kinetic constants, and their uncertainties, our method enhances the efficiency and accuracy of kinetic analysis, thus benefiting the wide community of researchers engaged in chemical kinetics studies.

The efficacy and accuracy of our approach have been demonstrated through various examples drawn from both educational contexts and recent literature in catalysis. We hope that the open‐access availability of IPLOT‐VKA Web Application will catalyze further advancements in kinetic analysis, opening new avenues for mechanistic investigations and contributing to the collective knowledge in the field of chemical kinetics.

## Methods

The mathematical derivation of the expansion of Powell‐Margerison method is fully discussed in the Supplementary Information. All the results and the plots presented in this work have been obtained through house‐made software which have been made freely accessible at the link (http://alelandi.pythonanywhere.com/) where a Tutorial Manual can also be found. Experimental times and concentrations for the cases discussed in this work are reported in the Supplementary Information, together with the references from which they have been retrieved.

## Supporting Information Summary

Mathematical derivation of equations discussed in the text. Analytical expressions of the integrated form of Equation (2), for different values of the parameters α and β . Additional plots for (i) other orders of reaction for the cases examined in Figures 1, 2, 4 and (ii) a further example on a dataset from ref. [4]. Tables reporting times and concentrations used in the kinetic analysis for the examples shown in this paper. A concise manual for our Web Application IPLOT‐VKA.

## Conflict of Interests

The authors declare no conflict of interest.

1

## Supporting information

As a service to our authors and readers, this journal provides supporting information supplied by the authors. Such materials are peer reviewed and may be re‐organized for online delivery, but are not copy‐edited or typeset. Technical support issues arising from supporting information (other than missing files) should be addressed to the authors.

Supporting Information

## Data Availability

The data that support the findings of this study are available in the supplementary material of this article.
