# Supplementary material for: IPLOT‐VKA: An Integral‐Method Powell‐pLOT‐Enhanced Visual Kinetic Analysis for the Determination of Orders of Reaction
Source: Chemistry. 2024 Dec 13;31(1):e202401914. doi: 10.1002/chem.202401914 (PMC11711304; doi:10.1002/chem.202401914)
Supplement: Supplementary file 1 — Supporting Information [file CHEM-31-e202401914-s001.pdf]

Chemistry–A European Journal

Supporting Information

IPLOT-VKA: An Integral-Method Powell-pLOT-Enhanced Visual Kinetic Analysis for the Determination of Orders of Reaction

Alessandro Landi* and Guglielmo Monaco*

Supplementary Information for: IPLOT-VKA: an Integral-method Powell-pLOT-enhanced Visual Kinetic Analysis for the determination of orders of reaction

Alessandro Landi* and Guglielmo Monaco*

Dipartimento di Chimica e Biologia Adolfo Zambelli, Università di Salerno, Via Giovanni Paolo II, I-84084 Fisciano (SA), Italy

E-mail: alelandi1@unisa.it; gmonaco@unisa.it

Contents

S1 Mathematical details of IPLOT-VKA method **S2**

S2 Analytical solutions of the integrals under study **S8**

S3 Disagreement between analytical and approximate solution **S17**

S4 Additional kinetic plots **S18**

S5 Residual standard errors **S25**

S6 Numerical data used for the kinetic analysis **S27**

S7 Manual of IPLOT-VKA Web-Application **S32**

References **S35**

,

S1 Mathematical details of IPLOT-VKA method

For a reaction

$$a\text{A} + b\text{B} \longrightarrow p\text{P} + q\text{Q}$$

the rate law is called *simple* if, in all the concentration domain spanned by experiments, it follows the equation

$$R = \frac{1}{\nu_\text{X}}\frac{d[\text{X}]}{dt} = k[\text{A}]^\alpha[\text{B}]^\beta \tag{S1}$$

for X any of A, B, P, Q, and $\nu_A = -a$, $\nu_B = -b$, $\nu_P = p$ and $\nu_Q = q$, or, equivalently,

$$R_A = -\frac{d[\text{A}]}{dt} = k_\text{A}[\text{A}]^\alpha[\text{B}]^\beta, \tag{S2}$$

with $k_A = ak$, and similar equations for other species.

It can be useful to consider Eq. S2 first in the basic case $\beta = 0$ and from two perspectives. First, as a differential equation, it can be solved numerically, as it is mandatory for most chemical systems. According to one of the simplest numerical methods, known as middle-point or second-order Runge-Kutta,[26] for $m+1$ time values t_0, $t_1 \ldots t_m$, and corresponding concentrations $[A]_0$, $[A]_i \ldots [A]_m$ and time intervals $\Delta t_i = t_i - t_{i-1}$, in order to get the concentration of P at time t, estimates are needed of the time derivatives of the rate R_A at the midpoint of the intervals. If the concentrations of A are already known at the chosen times, the needed estimates can be approximated as

$$[\text{P}] - [\text{P}]_0 = [\text{A}]_0 - [\text{A}] \simeq \Sigma_{i=1}^m R_A\left(t_i + \frac{\Delta t_i}{2}\right)\Delta t_i \simeq k_A \Sigma_{i=1}^m \left(\frac{[\text{A}]_i + [\text{A}]_{i-1}}{2}\right)^\alpha \Delta t_i, \tag{S3}$$

which can be recognized as the operative equation of the Variable Time Normalization Analysis (VTNA), in case of a single reactant.[4] Essentially, one computes different estimates of [P] for different values of the order α, and then chooses the order of reaction, which best matches the experimental values.

The very same strategy is shared from a second perspective, whereby the basic expression

Eq. S2 ($\beta = 0$) is integrated analytically. To this end, after separating the variables, it is useful to render adimensional the two sides by multiplying by a power of the initial concentration:

$$
\begin{aligned}
-[\mathrm{A}]_0^{\alpha-1} \int_{[\mathrm{A}]_0}^{[\mathrm{A}]} \frac{d[\mathrm{A}]}{[\mathrm{A}]^\alpha} \equiv - \int_1^\phi \frac{d\phi}{\phi^\alpha} \equiv \Delta f = \\
= kt[\mathrm{A}]_0^{\alpha-1} \equiv k\tau \equiv \theta,
\end{aligned}
\tag{S4}
$$

where we introduced $\phi = \frac{[\mathrm{A}]}{[\mathrm{A}]_0}$, i.e. the fraction of unreacted substrate, and an initial-concentration-weighted time τ, and the dimensionless θ, called reduced time by Margerison;[10] θ is the fractionation time if $kt^*[\mathrm{A}]_0^{\alpha-1} = 1$, where t^* is the unit of time (e.g. 1 s).

The definite integral

$$
\Delta f = f(1, \alpha) - f(\phi, \alpha) = \frac{1 - \phi^{1-\alpha}}{1 - \alpha} \tag{S5}
$$

can be straightforwardly computed and it is independent of initial concentrations (beware that for $\alpha = 1$, its limit form

$$
\lim_{\alpha \to 1} \Delta f = - \ln \phi \tag{S6}
$$

must be used).

Rather than plotting Δf vs t, as is typical in the integration method, Powell suggested to plot ϕ against the decimal logarithm of time, with the advantage that the value of ϕ of the experimental data is readily accessible. The relationship between ϕ and time can be worked out from Eq. S4 and S5 upon setting $k[A]_0^{1-\alpha} t^* = 1$:

$$
\phi = \left[1 - (1 - \alpha)\frac{t}{t^*}\right]^{\frac{1}{1-\alpha}}; \tag{S7}
$$

once more a limit form for $\alpha = 1$ is needed:

$$\lim_{\alpha \to 1} \ln \phi = -\frac{t}{t^*} \implies \phi = e^{-\frac{t}{t^*}} \tag{S8}$$

Coming back to the case of two reactants, the analysis of Eq. S2 as a simple reaction benefits from a check of whether the kinetic problem is one-dimensional, i.e. the stoichiometric coefficients are constant. In this case the disappearance of A and B are proportional to each other, $\frac{\Delta[\mathrm{B}]}{\Delta[\mathrm{A}]} = \frac{b}{a} = r$, and it is possible to write

$$[\mathrm{B}] = [\mathrm{B}]_0 - [\mathrm{A}]_0 r(1-\phi) = [\mathrm{A}]_0[I - r(1-\phi)], \tag{S9}$$

where $I = [\mathrm{B}]_0/[\mathrm{A}]_0$, so that the kinetic equation S2 can still be addressed by separation of variables ϕ and t.

In the special case of stoichiometric proportions of reactants, $I = \frac{b}{a} = r$, the rate law coincides with that of a single reactant with order $n = \alpha + \beta$ (the kinetic constant gets multiplied by r^β). Going to the general case, we get $I = r(1+c)$, where the parameter

$$c = \frac{[\mathrm{B}]_0}{[\mathrm{A}]_0}\frac{a}{b} - 1 \tag{S10}$$

is negative or positive if B is in stoichiometric defect or excess, respectively, over A.

Therefore, we can write $[\mathrm{B}] = [\mathrm{A}]_0\, r(\phi + c)$. At this point, by setting $\tau = t[\mathrm{A}]^{\alpha+\beta-1}$, separation of variables in Eq. S2 and multiplication of both sides by $[\mathrm{A}]_0^{\alpha+\beta-1}$ gives

$$-[\mathrm{A}]_0^{\alpha+\beta-1} \int_{[\mathrm{A}]_0}^{[\mathrm{A}]} \frac{d[\mathrm{A}]}{[\mathrm{A}]^\alpha [\mathrm{B}]^\beta} = k\tau \Rightarrow \tag{S11}$$

$$-\int_1^\phi \frac{d\phi}{\phi^\alpha(\phi+c)^\beta} = kr^\beta \tau \tag{S12}$$

Eventually, defining $-\int_1^\phi \frac{d\phi}{\phi^\alpha(\phi+c)^\beta} \equiv \Delta f(\phi;\alpha,\beta,c)$, we get:

$$\Delta f(\phi; \alpha, \beta, c) = kr^{\beta}\tau. \tag{S13}$$

Considering a change of variable, $\phi' = \phi + c$, integral $\Delta f(\phi; \alpha, \beta, c)$ can be written in two equivalent forms

$$\Delta f(\phi; \alpha, \beta, c) = -\int_1^{\phi} \frac{d\phi}{\phi^{\alpha}(\phi + c)^{\beta}} \tag{S14}$$

$$= -\int_{1+c}^{\phi+c} \frac{d\phi'}{(\phi' - c)^{\alpha}\phi'^{\beta}}, \tag{S15}$$

and each of them can be computed with a different primitive function:

$$\Delta f(\phi; \alpha, \beta, c) = -\int_1^{\phi} \frac{d\phi}{\phi^{\alpha}(\phi + c)^{\beta}} = f_I(1; \alpha, \beta, c) - f_I(\phi; \alpha, \beta, c) \tag{S16}$$

$$\Delta f(\phi; \alpha, \beta, c) = -\int_{1+c}^{\phi+c} \frac{d\phi'}{(\phi' - c)^{\alpha}\phi'^{\beta}} = f_{II}(1 + c; \alpha, \beta, c) - f_{II}(\phi + c; \alpha, \beta, c). \tag{S17}$$

Both primitive functions f_I and f_{II} are defined in terms of the hypergeometric function:

$$f_I(x; \alpha, \beta, c) = \frac{x^{1-\alpha}\,(c + x)^{-\beta}(1 + \frac{x}{c})^{\beta}\,{}_2\mathrm{F}_1\left(1 - \alpha, \beta;\ 2 - \alpha;\ -\frac{x}{c}\right)}{(1 - \alpha)}, \tag{S18}$$

$$f_{II}(x; \alpha, \beta, c) = \frac{x^{1-\beta}\,(x - c)^{-\alpha}(1 - \frac{x}{c})^{\alpha}\,{}_2\mathrm{F}_1\left(\alpha, 1 - \beta;\ 2 - \beta;\ \frac{x}{c}\right)}{(1 - \beta)}. \tag{S19}$$

The hypergeometric function is calculated by a power series, and coincides with common functions for special values of its parameters; however it also suffers from singularities for some values of its parameters.[24] The possibility to compute Δf by two different hypergeometric functions allows avoiding some of the singularities.

At semi-integer steps in the $[-1, 3]$ domain, 79 times out of 81, the primitive functions f_I

and/or f_{II} can be expressed as f_{III}, in terms of more common functions, so that

$$\Delta f(\phi;\alpha,\beta,c) = \Delta f_{III}(\phi;\alpha,\beta,c). \tag{S20}$$

15 of the Δf_{III} functions were already derived by Margerison,[10] while the remaining 64 solutions reported below have been derived by us for the first time and reported here, in Section S2. Some of Margerison's integrals had different analytic form for positive, negative or null values of c, but they all match exactly with those reported below.
The implementation of a code to compute Δf as a function of the four parameters α, β, c, ϕ has been performed in two ways in our Web-Application accessible at this link:

1. Use of f_I (Eq. S16) or f_{II} (Eq. S17) in the domain $\{(\alpha,\beta)\,|\,\alpha \in [-1,3] \wedge \beta \in [-1,3] - \Sigma_9\}$, which excludes the 9 singular points (those marked with a $-$ sign in the first two columns of Tables S1-S9). In a small neighbourhood of the singular points, Δf_{III} has been used.

2. Use of an approximation of Δf_{IV} of function Δf_{III} for all of the domain $\{(\alpha,\beta)\,|\,\alpha \in [-1,3] \wedge \beta \in [-1,3]\}$. Δf_{IV} coincides with Δf_{III} only at 79 points of the interval, in all other cases it is computed as

$$\begin{aligned}\Delta f_{IV}(\phi;\alpha',\beta',c) \simeq \quad & \Delta f_{III}(\phi;\alpha_i,\beta_i,c) \\ + \quad & \frac{\Delta f_{III}(\phi;\alpha_{i+1},\beta_i,c) - \Delta f_{III}(\phi;\alpha_i,\beta_i,c)}{\alpha_{i+1}-\alpha_i}(\alpha'-\alpha_i) \\ + \quad & \frac{\Delta f_{III}(\phi;\alpha_i,\beta_{i+1},c) - \Delta f_{III}(\phi;\alpha_i,\beta_i,c)}{\beta_{i+1}-\beta_i}(\beta'-\beta_i),\end{aligned} \tag{S21}$$

where $\alpha' \in [\alpha_i, \alpha_{i+1}]$ and $\beta' \in [\beta_i, \beta_{i+1}]$, and α_i and β_i are semi-integers where the function Δf_{III} is defined.

For both approaches, special care should be given close to $c = 0$, where some of the functions

are not defined; here, the following approximations can be used

$$-\frac{d\phi}{dt} \quad = \quad k_{\mathrm{A}}[\mathrm{A}]_0^{(\alpha+\beta-1)}\phi^{\alpha+\beta}r^{\beta}\left(1+\beta\frac{c}{\phi}\right) \qquad |c| << \phi$$

and then, considering that

$$\frac{1}{1+\beta\frac{c}{\phi}} \quad \simeq \quad 1-\beta\frac{c}{\phi} \qquad |\beta c| << \phi$$

$$\frac{\phi^{1-(\alpha+\beta)}-1}{1-(\alpha+\beta)} - \beta c\frac{\phi^{-(\alpha+\beta)}-1}{-(\alpha+\beta)} \quad = \quad -k_{\mathrm{A}}[\mathrm{A}]_0^{\alpha+\beta-1}r^{\beta}t \qquad |c| << \phi$$

The second approach (i.e. using Δf_{IV}) leads to errors, which are well acceptable for the discussion of partial orders. In the worst cases, obtained at the midpoint of the intervals between points where the function Δf_{IV} is exact, the disagreement factor

$$R^2 = \frac{\sum |\Delta f_{IV}(\phi_i;\alpha,\beta,c) - \Delta f(\phi_i;\alpha,\beta,c)|}{\sum \Delta f(\phi_i;\alpha,\beta,c)} \tag{S22}$$

is only a few percent points. The worst case over all, obtained for the unlikely total order of $n = \alpha + \beta = 5.5$ does not exceed the 7% (see Section S3).

S2 Analytical solutions of the integrals under study

In this section we report 9 Tables with the analytical expressions of integral of equation S13, for different values of the parameters α and β, in half-integer steps from -1.0 to 3.0

Table S1: Analytical expressions of integral in equation S13 for $\beta = -1$

α	Δf_I	Δf_{II}	Δf_{III}
-1	Eq. S16	Eq. S17	$\frac{x^2\,(3\,c+2\,x)}{6} - \frac{c}{2} - \frac{1}{3}$
-0.5	Eq. S16	Eq. S17	$\frac{2\,x^{3/2}\,(5\,c+3\,x)}{15} - \frac{2\,c}{3} - \frac{2}{5}$
0	Eq. S16	Eq. S17	$\frac{(x-1)\,(2\,c+x+1)}{2}$
0.5	Eq. S16	Eq. S17	$\frac{2\,\sqrt{x}\,(3\,c+x)}{3} - 2\,c - \frac{2}{3}$
1	–	Eq. S17	$x + c\,\ln(x) - 1$
1.5	Eq. S16	Eq. S17	$2\,c - \frac{2\,(c-x)}{\sqrt{x}} - 2$
2	–	Eq. S17	$c + \ln(x) - \frac{c}{x}$
2.5	Eq. S16	Eq. S17	$\frac{2\,c}{3} - \frac{2\,c+6\,x}{3\,x^{3/2}} + 2$
3	Eq. S16	Eq. S17	$\frac{c}{2} - \frac{c+2\,x}{2\,x^2} + 1$

Table S2: Analytical expressions of integral in equation S13 for $\beta = -0.5$

α	Δf_I	Δf_{II}	Δf_{III}
-1	Eq. S16	Eq. S17	$\frac{2\,(2\,c-3)\,(c+1)^{3/2}}{15} - \frac{2\,(c+x)^{3/2}\,(2\,c-3\,x)}{15}$
-0.5	Eq. S16	Eq. S17	$\frac{c^2\,\ln\left(c+2\,\sqrt{c+1}+2\right)}{8} - \left(\frac{c}{4}+\frac{1}{2}\right)\sqrt{c+1} - \frac{c^2\,\ln\left(c+2\,x+2\,\sqrt{x}\,\sqrt{c+x}\right)}{8} + \sqrt{x}\,\sqrt{c+x}\,\left(\frac{c}{4}+\frac{x}{2}\right)$
0	Eq. S16	Eq. S17	$\frac{2\,(c+x)^{3/2}}{3} - \frac{2\,(c+1)^{3/2}}{3}$
0.5	Eq. S16	Eq. S17	$\sqrt{x}\,\sqrt{c+x} - 2\,c\,\mathrm{atanh}\left(\frac{1}{\sqrt{c+1}-\sqrt{c}}\right) - \sqrt{c+1} + 2\,c\,\mathrm{atanh}\left(\frac{\sqrt{x}}{\sqrt{c+x}-\sqrt{c}}\right)$
1	–	Eq. S17	$2\,\sqrt{c+x} - 2\,\sqrt{c}\,\mathrm{atanh}\left(\frac{\sqrt{c+x}}{\sqrt{c}}\right) - 2\,\sqrt{c+1} + 2\,\sqrt{c}\,\mathrm{atanh}\left(\frac{\sqrt{c+1}}{\sqrt{c}}\right)$
1.5	Eq. S16	Eq. S17	–
2	–	Eq. S17	$\sqrt{c+1} - \frac{\mathrm{atanh}\left(\frac{\sqrt{c+x}}{\sqrt{c}}\right)}{\sqrt{c}} + \frac{\mathrm{atanh}\left(\frac{\sqrt{c+1}}{\sqrt{c}}\right)}{\sqrt{c}} - \frac{\sqrt{c+x}}{x}$
2.5	Eq. S16	Eq. S17	$\frac{2\,(c+1)^{3/2}}{3\,c} - \frac{2\,(c+x)^{3/2}}{3\,c\,x^{3/2}}$
3	–	Eq. S17	$\frac{\sqrt{c+1}}{4} + \frac{\mathrm{atanh}\left(\frac{\sqrt{c+x}}{\sqrt{c}}\right)}{4\,c^{3/2}} + \frac{(c+1)^{3/2}}{4\,c} - \frac{\mathrm{atanh}\left(\frac{\sqrt{c+1}}{\sqrt{c}}\right)}{4\,c^{3/2}} - \frac{\sqrt{c+x}}{4\,x^2} - \frac{(c+x)^{3/2}}{4\,c\,x^2}$

Table S3: Analytical expressions of integral in equation S13 for $\beta = 0$

α	Δf_I	Δf_{II}	Δf_{III}
-1	Eq. S16	Eq. S17	$\frac{x^2}{2} - \frac{1}{2}$
-0.5	Eq. S16	Eq. S17	$\frac{2\,x^{3/2}}{3} - \frac{2}{3}$
0	Eq. S16	Eq. S17	$x - 1$
0.5	Eq. S16	Eq. S17	$2\,\sqrt{x} - 2$
1	–	Eq. S17	$\ln(x)$
1.5	Eq. S16	Eq. S17	$2 - \frac{2}{\sqrt{x}}$
2	Eq. S16	Eq. S17	$1 - \frac{1}{x}$
2.5	Eq. S16	Eq. S17	$\frac{2}{3} - \frac{2}{3\,x^{3/2}}$
3	Eq. S16	Eq. S17	$\frac{1}{2} - \frac{1}{2\,x^2}$

Table S4: Analytical expressions of integral in equation S13 for $\beta = 0.5$

α	Δf_I	Δf_{II}	Δf_{III}
-1	Eq. S16	Eq. S17	$\frac{2\,(2\,c-1)\,\sqrt{c+1}}{3} - \frac{2\,\sqrt{c+x}\,(2\,c-x)}{3}$
-0.5	Eq. S16	Eq. S17	$2\,c\,\mathrm{atanh}\left(\frac{1}{\sqrt{c+1}-\sqrt{c}}\right) - \sqrt{c+1} + \sqrt{x}\,\sqrt{c+x} - 2\,c\,\mathrm{atanh}\left(\frac{\sqrt{x}}{\sqrt{c+x}-\sqrt{c}}\right)$
0	Eq. S16	Eq. S17	$2\,\sqrt{c+x} - 2\,\sqrt{c+1}$
0.5	Eq. S16	Eq. S17	$4\,\mathrm{atanh}\left(\frac{\sqrt{c+x}-\sqrt{c}}{\sqrt{x}}\right) - 4\,\mathrm{atanh}\left(\sqrt{c+1}-\sqrt{c}\right)$
1	–	Eq. S17	$\frac{2\,\mathrm{atanh}\left(\frac{\sqrt{c+1}}{\sqrt{c}}\right)}{\sqrt{c}} - \frac{2\,\mathrm{atanh}\left(\frac{\sqrt{c+x}}{\sqrt{c}}\right)}{\sqrt{c}}$
1.5	Eq. S16	Eq. S17	$\frac{2\,\sqrt{c+1}}{c} - \frac{2\,\sqrt{c+x}}{c\,\sqrt{x}}$
2	–	Eq. S17	$\frac{\mathrm{atanh}\left(\frac{\sqrt{c+x}}{\sqrt{c}}\right)}{c^{3/2}} + \frac{\sqrt{c+1}}{c} - \frac{\mathrm{atanh}\left(\frac{\sqrt{c+1}}{\sqrt{c}}\right)}{c^{3/2}} - \frac{\sqrt{c+x}}{c\,x}$
2.5	Eq. S16	Eq. S17	$\frac{2\,\sqrt{c+1}\,(c-2)}{3\,c^2} - \frac{2\,\sqrt{c+x}\,(c-2\,x)}{3\,c^2\,x^{3/2}}$
3	–	Eq. S17	$\frac{5\,\sqrt{c+1}}{4\,c} - \frac{3\,\mathrm{atanh}\left(\frac{\sqrt{c+x}}{\sqrt{c}}\right)}{4\,c^{5/2}} - \frac{3\,(c+1)^{3/2}}{4\,c^2} + \frac{3\,\mathrm{atanh}\left(\frac{\sqrt{c+1}}{\sqrt{c}}\right)}{4\,c^{5/2}} - \frac{5\,\sqrt{c+x}}{4\,c\,x^2} + \frac{3\,(c+x)^{3/2}}{4\,c^2\,x^2}$

Table S5: Analytical expressions of integral in equation S13 for $\beta = 1$

α	Δf_I	Δf_{II}	Δf_{III}
-1	Eq. S16	Eq. S17	$x - c\,\ln(c+x) + c\,\ln(c+1) - 1$
-0.5	Eq. S16	Eq. S17	$2\sqrt{c}\left(\mathrm{atan}\left(\frac{1}{\sqrt{c}}\right) - \mathrm{atan}\left(\frac{\sqrt{x}}{\sqrt{c}}\right)\right) + 2\sqrt{x} - 2$
0	Eq. S16	Eq. S17	$\ln(c+x) - \ln(c+1)$
0.5	Eq. S16	Eq. S17	$-\frac{2\left(\mathrm{atan}\left(\frac{1}{\sqrt{c}}\right) - \mathrm{atan}\left(\frac{\sqrt{x}}{\sqrt{c}}\right)\right)}{\sqrt{c}}$
1	–	–	$\frac{\ln(c+1) - \ln\left(\frac{c}{x}+1\right)}{c}$
1.5	Eq. S16	Eq. S17	$\frac{2\left(\mathrm{atan}\left(\frac{1}{\sqrt{c}}\right) - \mathrm{atan}\left(\frac{\sqrt{x}}{\sqrt{c}}\right)\right)}{c^{3/2}} + \frac{2}{c} - \frac{2}{c\sqrt{x}}$
2	–	–	$-\frac{c - c\,x - x\,\ln\left(\frac{1}{c+1}\right) + x\,\ln\left(\frac{x}{c+x}\right)}{c^2\,x}$
2.5	Eq. S16	Eq. S17	$\frac{2}{3\,c} - \frac{2\left(\mathrm{atan}\left(\frac{1}{\sqrt{c}}\right) - \mathrm{atan}\left(\frac{\sqrt{x}}{\sqrt{c}}\right)\right)}{c^{5/2}} - \frac{2}{c^2} - \frac{2\,c - 6\,x}{3\,c^2\,x^{3/2}}$
3	–	–	$\frac{2\,\mathrm{atanh}\left(\frac{2}{c}+1\right)}{c^3} - \frac{c - \frac{c^2}{2}}{c^3} - \frac{2\,\mathrm{atanh}\left(\frac{2\,x}{c}+1\right)}{c^3} + \frac{c\,x - \frac{c^2}{2}}{c^3\,x^2}$

Table S6: Analytical expressions of integral in equation S13 for $\beta = 1.5$

α	Δf_I	Δf_{II}	Δf_{III}
-1	Eq. S16	Eq. S17	$\frac{4\,c+2\,x}{\sqrt{c+x}} - \frac{4\,c+2}{\sqrt{c+1}}$
-0.5	Eq. S16	Eq. S17	–
0	Eq. S16	Eq. S17	$\frac{2}{\sqrt{c+1}} - \frac{2}{\sqrt{c+x}}$
0.5	Eq. S16	Eq. S17	$\frac{2\,\sqrt{x}}{c\,\sqrt{c+x}} - \frac{2}{c\,\sqrt{c+1}}$
1	–	Eq. S17	$\frac{2}{c\,\sqrt{c+x}} - \frac{2}{c\,\sqrt{c+1}} - \frac{2\,\mathrm{atanh}\left(\frac{\sqrt{c+x}}{\sqrt{c}}\right)}{c^{3/2}} + \frac{2\,\mathrm{atanh}\left(\frac{\sqrt{c+1}}{\sqrt{c}}\right)}{c^{3/2}}$
1.5	Eq. S16	Eq. S17	$\frac{2\,(c+2)}{c^2\,\sqrt{c+1}} - \frac{2\,(c+2\,x)}{c^2\,\sqrt{x}\,\sqrt{c+x}}$
2	–	Eq. S17	$\frac{3\,\mathrm{atanh}\left(\frac{\sqrt{c+x}}{\sqrt{c}}\right)}{c^{5/2}} - \frac{3\,\mathrm{atanh}\left(\frac{\sqrt{c+1}}{\sqrt{c}}\right)}{c^{5/2}} + \frac{c+3}{c^2\,\sqrt{c+1}} - \frac{c+3\,x}{c^2\,x\,\sqrt{c+x}}$
2.5	Eq. S16	Eq. S17	$\frac{2\left(-c^2+4\,c\,x+8\,x^2\right)}{3\,c^3\,x^{3/2}\,\sqrt{c+x}} - \frac{-2\,c^2+8\,c+16}{3\,c^3\,\sqrt{c+1}}$
3	–	Eq. S17	$\frac{15\,\mathrm{atanh}\left(\frac{\sqrt{c+1}}{\sqrt{c}}\right)}{4\,c^{7/2}} - \frac{15\,\mathrm{atanh}\left(\frac{\sqrt{c+x}}{\sqrt{c}}\right)}{4\,c^{7/2}} - \frac{-2\,c^2+5\,c+15}{4\,c^3\,\sqrt{c+1}} + \frac{-2\,c^2+5\,c\,x+15\,x^2}{4\,c^3\,x^2\,\sqrt{c+x}}$

Table S7: Analytical expressions of integral in equation S13 for $\beta = 2$

α	Δf_I	Δf_{II}	Δf_{III}
-1	Eq. S16	–	$\ln(c+x) - \ln(c+1) + \frac{c}{c+x} - \frac{c}{c+1}$
-0.5	Eq. S16	–	$\frac{1}{c+1} - \frac{\mathrm{atan}\left(\frac{1}{\sqrt{c}}\right) - \mathrm{atan}\left(\frac{\sqrt{x}}{\sqrt{c}}\right)}{\sqrt{c}} - \frac{\sqrt{x}}{c+x}$
0	Eq. S16	Eq. S17	$\frac{1}{c+1} - \frac{1}{c+x}$
0.5	Eq. S16	–	$\frac{\sqrt{x}}{c\,(c+x)} - \frac{\mathrm{atan}\left(\frac{1}{\sqrt{c}}\right) - \mathrm{atan}\left(\frac{\sqrt{x}}{\sqrt{c}}\right)}{c^{3/2}} - \frac{1}{c\,(c+1)}$
1	–	–	$\frac{\ln(c+1)}{c^2} - \frac{1}{c\,(c+1)} + \frac{1}{c\,(c+x)} - \frac{\ln\left(\frac{c+x}{x}\right)}{c^2}$
1.5	Eq. S16	–	$\frac{3\left(\mathrm{atan}\left(\frac{1}{\sqrt{c}}\right) - \mathrm{atan}\left(\frac{\sqrt{x}}{\sqrt{c}}\right)\right)}{c^{5/2}} + \frac{2\,c+3}{c^2\,(c+1)} - \frac{2\,c+3\,x}{c^2\,\sqrt{x}\,(c+x)}$
2	–	–	$\frac{1}{c\,(c+1)} + \frac{2}{c^2\,(c+1)} - \frac{2\,\ln(c+1)}{c^3} - \frac{2}{c^2\,(c+x)} + \frac{2\,\ln\left(\frac{c+x}{x}\right)}{c^3} - \frac{1}{c\,x\,(c+x)}$
2.5	Eq. S16	–	$\frac{-2\,c^2+10\,c\,x+15\,x^2}{3\,c^3\,x^{3/2}\,(c+x)} - \frac{-2\,c^2+10\,c+15}{3\,c^3\,(c+1)} - \frac{5\left(\mathrm{atan}\left(\frac{1}{\sqrt{c}}\right) - \mathrm{atan}\left(\frac{\sqrt{x}}{\sqrt{c}}\right)\right)}{c^{7/2}}$
3	–	–	$\frac{6\,\mathrm{atanh}\left(\frac{c+2}{c}\right)}{c^4} - \frac{6\,\mathrm{atanh}\left(\frac{c+2\,x}{c}\right)}{c^4} - \frac{-c^2+3\,c+6}{2\,c^3\,(c+1)} + \frac{-c^2+3\,c\,x+6\,x^2}{2\,c^3\,x^2\,(c+x)}$

Table S8: Analytical expressions of integral in equation S13 for $\beta = 2.5$

α	Δf_I	Δf_{II}	Δf_{III}
-1	Eq. S16	Eq. S17	$\frac{4\,c+6}{3\,(c+1)^{3/2}} - \frac{4\,c+6\,x}{3\,(c+x)^{3/2}}$
-0.5	Eq. S16	Eq. S17	$\frac{2\,x^{3/2}}{3\,c\,(c+x)^{3/2}} - \frac{2}{3\,c\,(c+1)^{3/2}}$
0	Eq. S16	Eq. S17	$\frac{2}{3\,(c+1)^{3/2}} - \frac{2}{3\,(c+x)^{3/2}}$
0.5	Eq. S16	Eq. S17	$\frac{2\,\sqrt{x}\,(3\,c+2\,x)}{3\,c^2\,(c+x)^{3/2}} - \frac{2\,(3\,c+2)}{3\,c^2\,(c+1)^{3/2}}$
1	–	Eq. S17	$\frac{2\,\mathrm{atanh}\left(\frac{\sqrt{c+1}}{\sqrt{c}}\right)}{c^{5/2}} - \frac{2\,\mathrm{atanh}\left(\frac{\sqrt{c+x}}{\sqrt{c}}\right)}{c^{5/2}} - \frac{8\,c+6}{3\,c^2\,(c+1)^{3/2}} + \frac{8\,c+6\,x}{3\,c^2\,(c+x)^{3/2}}$
1.5	Eq. S16	Eq. S17	$\frac{2\left(3\,c^2+12\,c+8\right)}{3\,c^3\,(c+1)^{3/2}} - \frac{2\left(3\,c^2+12\,c\,x+8\,x^2\right)}{3\,c^3\,\sqrt{x}\,(c+x)^{3/2}}$
2	–	Eq. S17	$\frac{5\,\mathrm{atanh}\left(\frac{\sqrt{c+x}}{\sqrt{c}}\right)}{c^{7/2}} - \frac{5\,\mathrm{atanh}\left(\frac{\sqrt{c+1}}{\sqrt{c}}\right)}{c^{7/2}} + \frac{3\,c^2+20\,c+15}{3\,c^3\,(c+1)^{3/2}} - \frac{3\,c^2+20\,c\,x+15\,x^2}{3\,c^3\,x\,(c+x)^{3/2}}$
2.5	Eq. S16	Eq. S17	$\frac{2\left(-c^3+6\,c^2\,x+24\,c\,x^2+16\,x^3\right)}{3\,c^4\,x^{3/2}\,(c+x)^{3/2}} - \frac{2\left(-c^3+6\,c^2+24\,c+16\right)}{3\,c^4\,(c+1)^{3/2}}$
3	–	Eq. S17	$\frac{35\,\mathrm{atanh}\left(\frac{\sqrt{c+1}}{\sqrt{c}}\right)}{4\,c^{9/2}} - \frac{35\,\mathrm{atanh}\left(\frac{\sqrt{c+x}}{\sqrt{c}}\right)}{4\,c^{9/2}} - \frac{-6\,c^3+21\,c^2+140\,c+105}{12\,c^4\,(c+1)^{3/2}} + \frac{-6\,c^3+21\,c^2\,x+140\,c\,x^2+105\,x^3}{12\,c^4\,x^2\,(c+x)^{3/2}}$

Table S9: Analytical expressions of integral in equation S13 for $\beta = 3$

α	Δf_I	Δf_{II}	Δf_{III}
-1	Eq. S16	Eq. S17	$\frac{(x-1)\,(c+2\,x+c\,x)}{2\,(c+x)^2\,(c+1)^2}$
-0.5	Eq. S16	–	$\frac{c-1}{4\,c\,(c+1)^2} - \frac{\mathrm{atan}\left(\frac{1}{\sqrt{c}}\right)-\mathrm{atan}\left(\frac{\sqrt{x}}{\sqrt{c}}\right)}{4\,c^{3/2}} - \frac{\sqrt{x}\,(c-x)}{4\,c\,(c+x)^2}$
0	Eq. S16	Eq. S17	$\frac{1}{2\,(c+1)^2} - \frac{1}{2\,(c+x)^2}$
0.5	Eq. S16	–	$\frac{\sqrt{x}\,(5\,c+3\,x)}{4\,c^2\,(c+x)^2} - \frac{5\,c+3}{4\,c^2\,(c+1)^2} - \frac{3\left(\mathrm{atan}\left(\frac{1}{\sqrt{c}}\right)-\mathrm{atan}\left(\frac{\sqrt{x}}{\sqrt{c}}\right)\right)}{4\,c^{5/2}}$
1	–	–	$\frac{\ln(c+1)+\frac{2}{c+1}-\frac{1}{2\,(c+1)^2}}{c^3} - \frac{\ln\left(\frac{c+x}{x}\right)+\frac{2\,x}{c+x}-\frac{x^2}{2\,(c+x)^2}}{c^3}$
1.5	Eq. S16	–	$\frac{15\left(\mathrm{atan}\left(\frac{1}{\sqrt{c}}\right)-\mathrm{atan}\left(\frac{\sqrt{x}}{\sqrt{c}}\right)\right)}{4\,c^{7/2}} + \frac{8\,c^2+25\,c+15}{4\,c^3\,(c+1)^2} - \frac{8\,c^2+25\,c\,x+15\,x^2}{4\,c^3\,\sqrt{x}\,(c+x)^2}$
2	–	–	$\frac{6\,\mathrm{atanh}\left(\frac{c+2\,x}{c}\right)}{c^4} - \frac{6\,\mathrm{atanh}\left(\frac{c+2}{c}\right)}{c^4} + \frac{2\,c^2+9\,c+6}{2\,c^3\,(c+1)^2} - \frac{2\,c^2+9\,c\,x+6\,x^2}{2\,c^3\,x\,(c+x)^2}$
2.5	Eq. S16	–	$\frac{-8\,c^3+56\,c^2\,x+175\,c\,x^2+105\,x^3}{12\,c^4\,x^{3/2}\,(c+x)^2} - \frac{-8\,c^3+56\,c^2+175\,c+105}{12\,c^4\,(c+1)^2} - \frac{35\left(\mathrm{atan}\left(\frac{1}{\sqrt{c}}\right)-\mathrm{atan}\left(\frac{\sqrt{x}}{\sqrt{c}}\right)\right)}{4\,c^{9/2}}$
3	–	–	$\frac{12\,\mathrm{atanh}\left(\frac{c+2}{c}\right)}{c^5} - \frac{12\,\mathrm{atanh}\left(\frac{c+2\,x}{c}\right)}{c^5} - \frac{-c^3+4\,c^2+18\,c+12}{2\,c^4\,(c+1)^2} + \frac{-c^3+4\,c^2\,x+18\,c\,x^2+12\,x^3}{2\,c^4\,x^2\,(c+x)^2}$

S3 Disagreement between analytical and approximate solution

In this section we report the disagreement factor between Δf_{III} and exact (based on hypergeometric) solutions discussed in Section S1, defined as

$$R^2 = \frac{\sum |\Delta f_{III}(\phi_i; \alpha, \beta, c) - \Delta f(\phi_i; \alpha, \beta, c)|}{\sum \Delta f(\phi_i; \alpha, \beta, c)} \tag{S23}$$

for the worst cases, i.e. at the midpoint of the intervals between points where the function Δf_{III} is exact.

Table S10: Disagreement factor R^2, eq. S23, computed for a vector of 9 elements $\boldsymbol{\phi} = (0.1, 0.2, 0.3, 0.4, 0.5, 0.6, 0.7, 0.8, 0.9)$ and $c = 0.5$.

α \ β	-0.75	-0.25	0.25	0.75	1.25	1.75	2.25	2.75
-0.75	0.009	0.010	0.011	0.011	0.012	0.032	0.014	0.016
-0.25	0.011	0.011	0.012	0.013	0.028	0.016	0.018	0.019
0.25	0.012	0.014	0.015	0.016	0.018	0.020	0.022	0.025
0.75	0.015	0.017	0.019	0.021	0.023	0.026	0.029	0.031
1.25	0.020	0.066	0.025	0.027	0.030	0.033	0.036	0.040
1.75	0.055	0.029	0.032	0.036	0.039	0.042	0.045	0.048
2.25	0.034	0.038	0.041	0.045	0.048	0.051	0.054	0.056
2.75	0.044	0.048	0.051	0.054	0.057	0.059	0.061	0.064

Table S11: Disagreement factor R^2, eq. S23, computed for a vector of 9 elements $\boldsymbol{\phi} = (0.1, 0.2, 0.3, 0.4, 0.5, 0.6, 0.7, 0.8, 0.9)$ and $c = -0.5$.

α \ β	-0.75	-0.25	0.25	0.75	1.25	1.75	2.25	2.75
-0.75	0.025	0.027	0.029	0.033	0.037	0.044	0.048	0.054
-0.25	0.025	0.027	0.030	0.034	0.028	0.044	0.050	0.056
0.25	0.026	0.028	0.031	0.035	0.040	0.046	0.052	0.058
0.75	0.027	0.029	0.033	0.037	0.042	0.048	0.054	0.060
1.25	0.028	0.067	0.034	0.038	0.044	0.049	0.056	0.062
1.75	0.086	0.031	0.035	0.040	0.045	0.051	0.057	0.063
2.25	0.029	0.033	0.037	0.042	0.047	0.053	0.059	0.065
2.75	0.030	0.034	0.038	0.043	0.049	0.055	0.061	0.066

S4 Additional kinetic plots

In this section, we report additional kinetic plots for the cases discussed in the main text. In particular,

- Fig. S1 shows that, analyzing the kinetic data reported in ref. 21 (Fig. 1 of the main text), the fitting is unsatisfactory for orders different from 2.
- Fig. S2 shows that, analyzing the kinetic data reported in ref. 23 (Fig.2 of the main text), the fitting for order 0.6 turns out preferable with respect to 0.5 (see Main text). However, as discussed in section S5, the lowering of the residual standard deviation is not statistically significant.
- Fig. S3 shows that, analyzing the kinetic data discussed in Fig. 2 of ref. 4 (Fig. 4 of the main text), the fitting is unsatisfactory for orders different from 2.
- Fig. S4 reports the scheme of the reaction discussed in Fig. 4 of ref. 4
- Fig. S5 shows the systematic kinetic analysis for the reaction reported in Fig. S4
- Fig. S6 shows the systematic kinetic analysis for the reaction reported in Fig. S4 using only some of the datasets, at variance with Fig. S5, where all three dataset have been used.

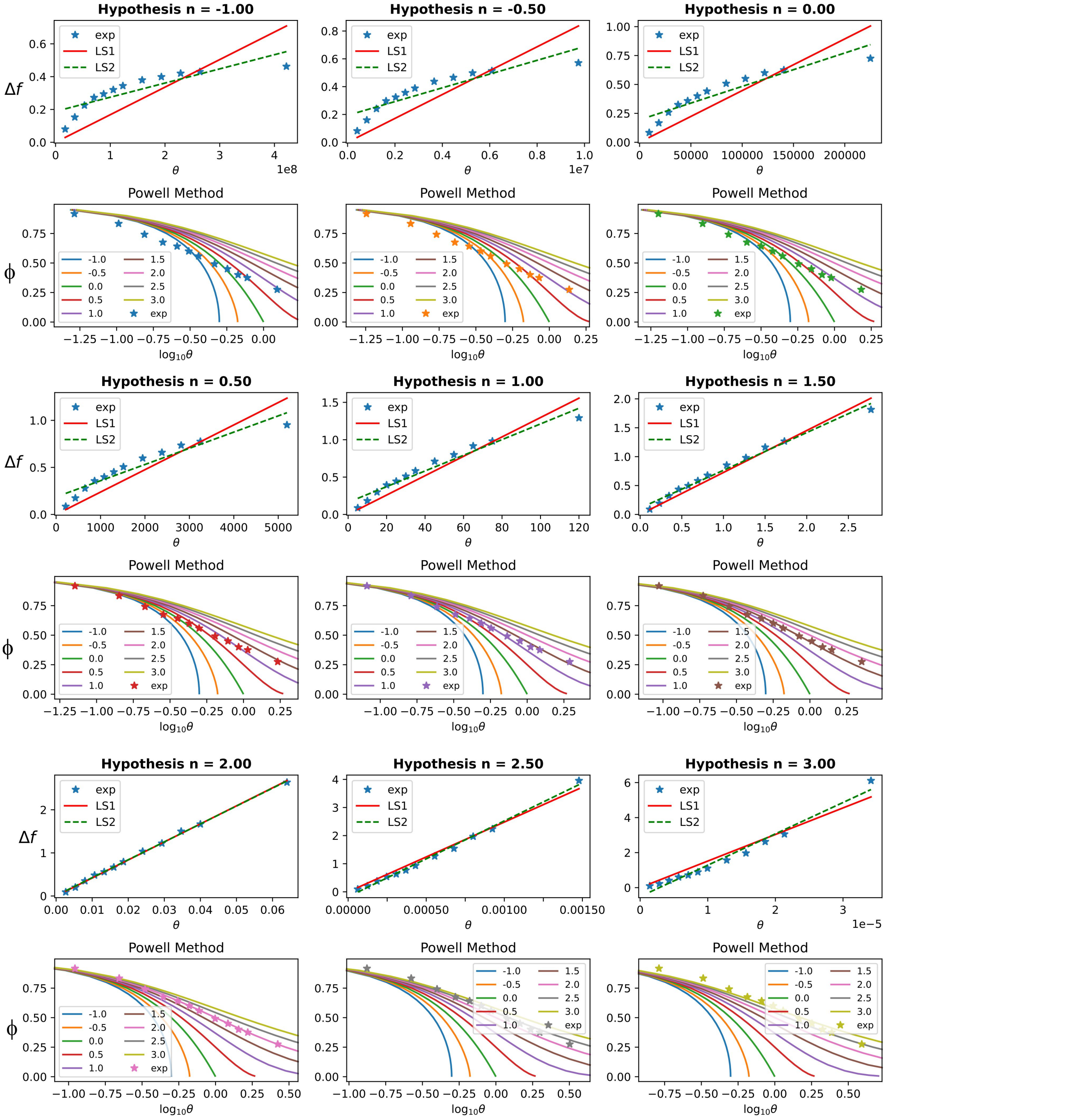

Figure S1: The Powell method applied to the kinetic data reported in ref. 21, showing that, for orders different from 2, the fitting is unsatisfactory.

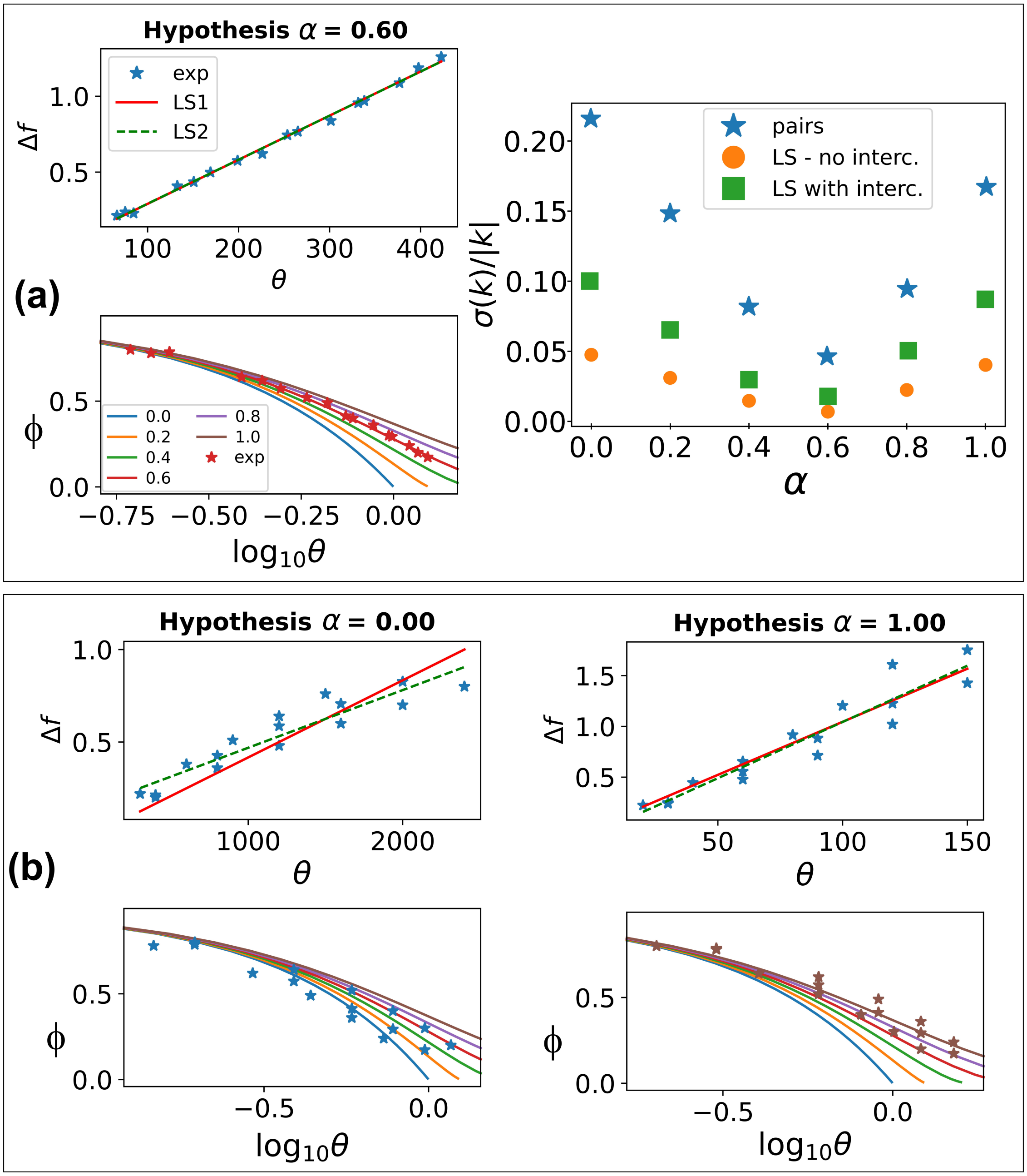

Figure S2: The Powell method applied to the kinetic data reported in ref. 27, showing the fitting for order 0.6

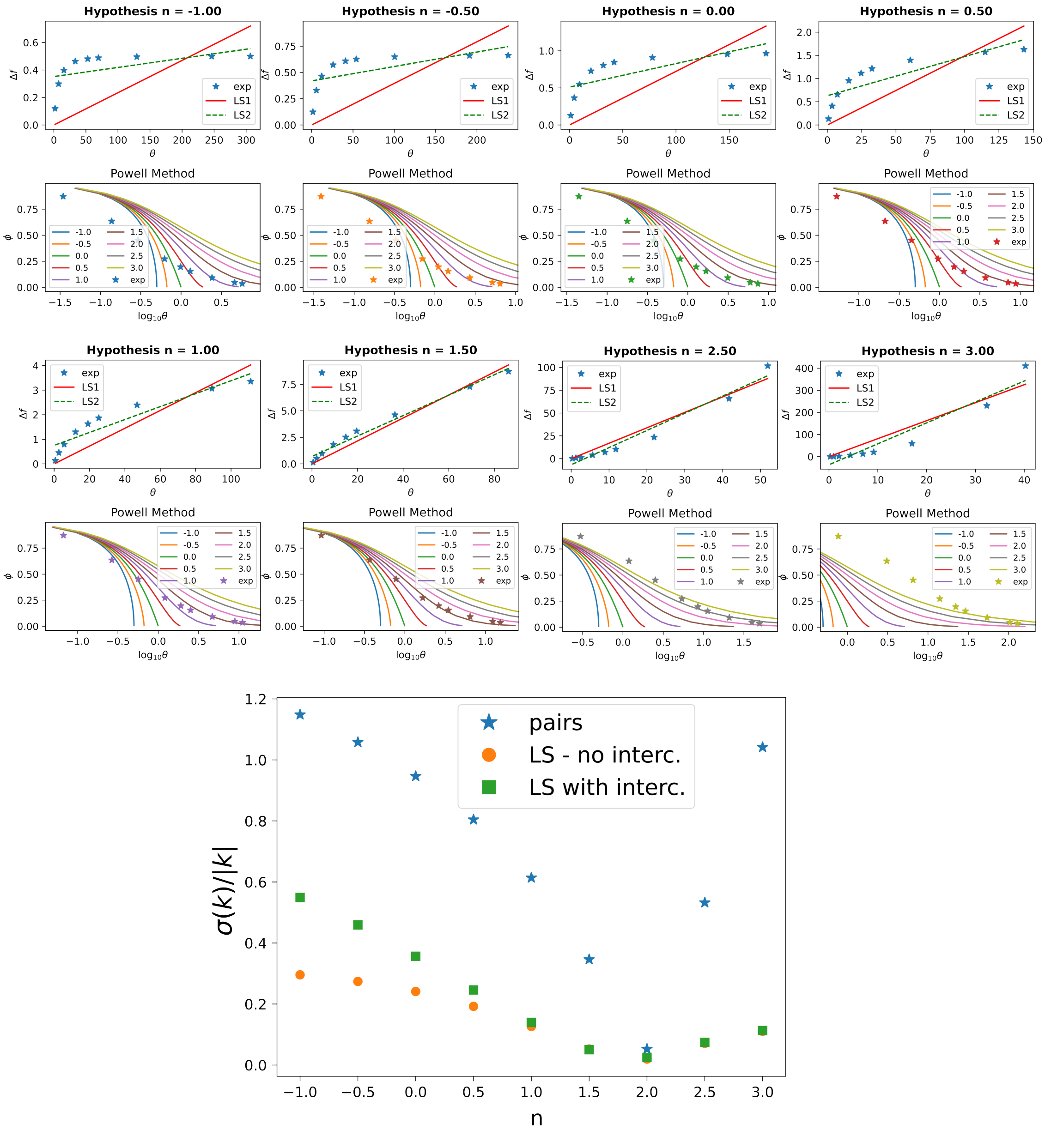

Figure S3: The Powell method applied to the kinetic data reported in Fig. 2 of ref. 4, showing that, for orders different from 2, the fitting is unsatisfactory.

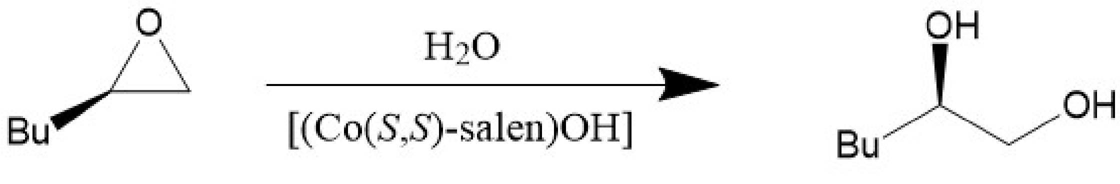

5

6

Figure S4: Scheme of the reaction reported in ref. 28.

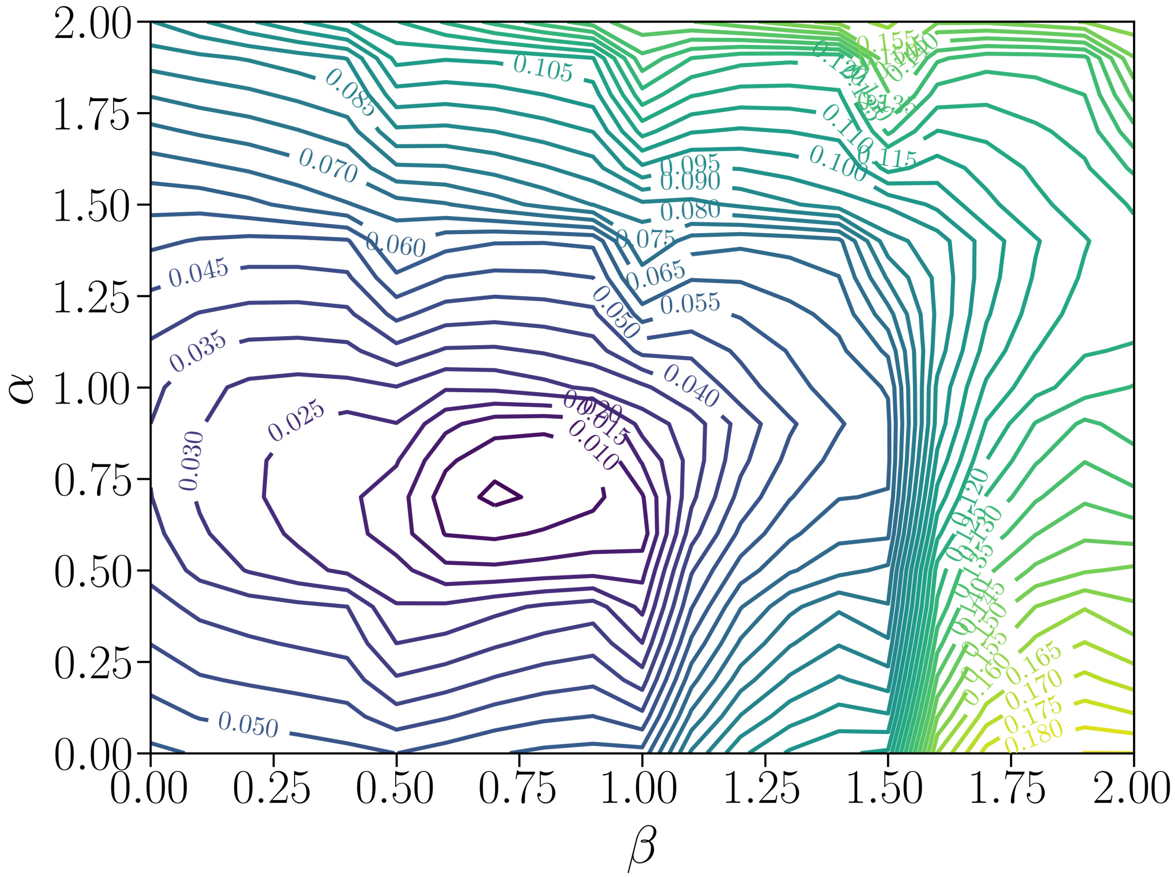

Figure S5: Contours of relative error on the kinetic constant as a function of the two partial orders α and β, for the reaction reported in Fig. S4 (Kinetic data retrieved from Section 6.3 of the Supporting Information of ref. 4.)

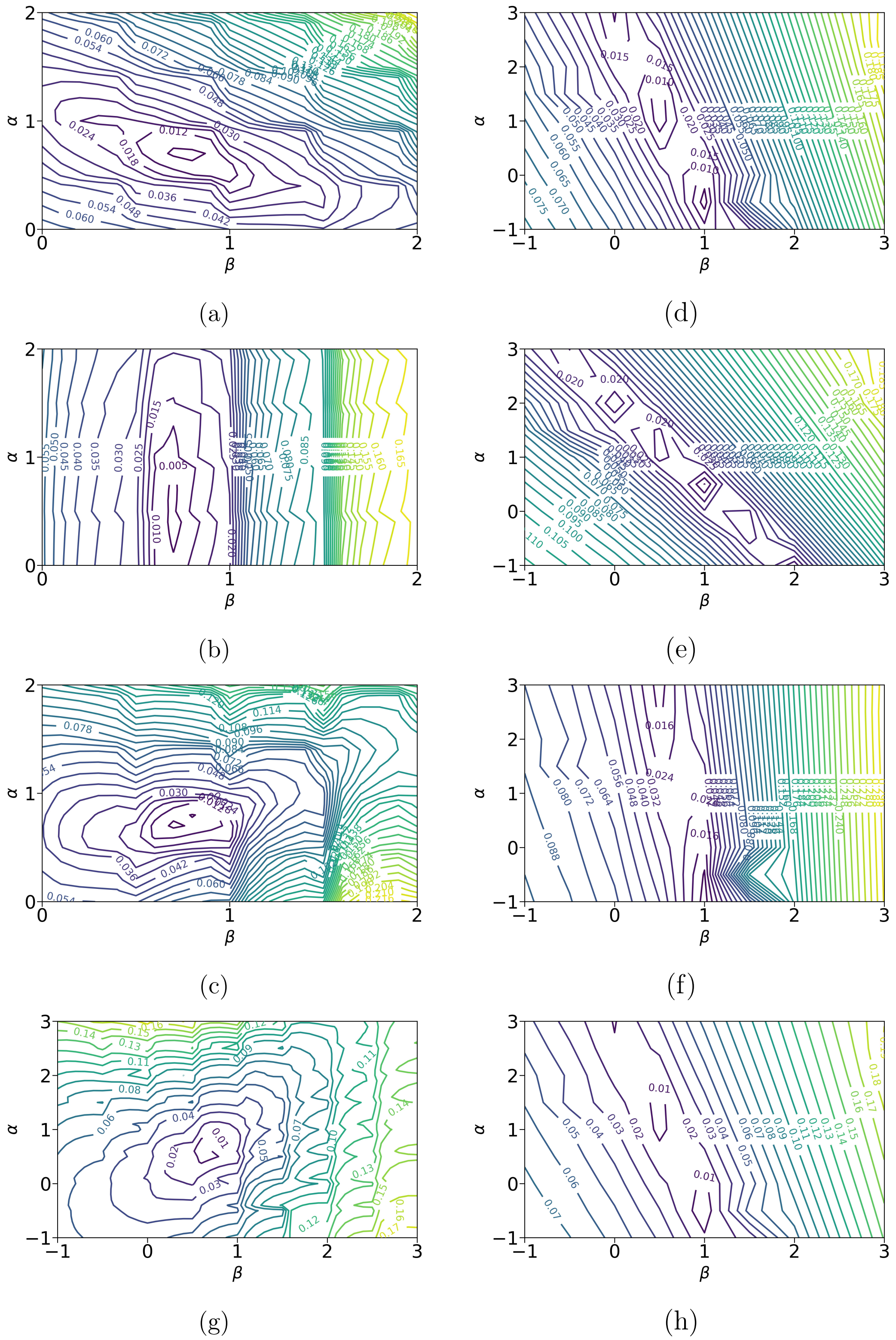

Figure S6: Contours of relative error on the kinetic constant as a function of the two partial orders α and β, for the reaction reported in Fig. S4. In panels a-f the kinetic data have been retrieved from Section 6.3 of the Supporting Information of ref. 4: (a) sets 1 and 2, (b) sets 1 and 3, (c) sets 2 and 3; (d) set 1, (e) set 2, (f) set 3. In panels g-h the kinetic data have been generated for this work and are detailed in Table S18: panel g is based on set 1 and 2, while panel h is based on set 1 only.

S5 Residual standard errors

In this section, we discuss the use of residual standard error on concentration

$$s([\mathrm{A}]_{\mathrm{exp}} - [\mathrm{A}]_{\mathrm{calc}}) = \sqrt{\frac{\sum([\mathrm{A}]_{\mathrm{exp},i} - [\mathrm{A}]_{\mathrm{calc},i})^2}{\nu}}, \tag{S24}$$

where ν represents the number of degrees of freedom, given by:

$$\nu = \nu_A - \nu_{A0} - 1 \tag{S25}$$

where ν_A is the number of data (concentrations) used in the kinetic analysis, ν_{A0} is the number of data (concentrations) measured at $t = 0$ and finally the -1 stems from the single parameter fitted (the kinetic constant).

To understand if the change in the goodness of fit (i.e. the change in $s([\mathrm{A}]_{\mathrm{exp}} - [\mathrm{A}]_{\mathrm{calc}})$) obtained using a finer grid (e.g. passing from a 0.5-step grid to a 0.1-step grid) for the orders of reaction is significant, the F-test can be used. To this end we compute the F value as the ratio of the two estimated variances,

$$F = \frac{s_{0.1}^2}{s_{0.5}^2} \tag{S26}$$

If $F < 1$, the finer grid leads to lowering the residual standard error, but this lowering is considered significant at the 5% level if $F < F_{\mathrm{crit}}(0.05, \nu, \nu)$, where $F_{\mathrm{crit}}(0.05, \nu_1, \nu_2)$ is the critical value, reported in statistical tables and computed by many statistical software, such that the cumulative F distribution $F(F_{\mathrm{crit}}, \nu_1, \nu_2) = 0.05$.

For the cases under study such a statistical analysis has been performed, obtaining the following results:

- **Fig. 2 of main text:** Residual standard error on concentration $s([\mathrm{A}]_{\mathrm{exp}} - [\mathrm{A}]_{\mathrm{calc}})$ are 1.07×10^{-3} M and 1.04×10^{-3} M, for the orders $\alpha = 0.5$ and $\alpha = 0.6$, respectively. The number of degrees of freedom is 15. Since the F-ratio (Eq. S24) $F = 0.943$ is not lower than the tabulated $F(0.05, 15, 15) = 0.416$, the reduction of the variance is not

significant at the 5% level, and the introduction of a non-half-integer partial order is not statistically justified.

- **Fig. 3 of main text:** Residual standard error on concentration $s([\mathrm{A}]_{\mathrm{exp}} - [\mathrm{A}]_{\mathrm{calc}})$ are 1.42×10^{-4} M and 1.54×10^{-4} M, for partial orders (α, β) equal to (1.0,1.0) and (1.1,0.9), respectively. In this case we even have that F-ratio is higher than 1 ($F = 1.08$), which means that there is no reduction of the variance, therefore the introduction of a non-half-integer partial order is not statistically justified.

- **Fig. S5:** Residual standard error on concentration $s([\mathrm{A}]_{\mathrm{exp}} - [\mathrm{A}]_{\mathrm{calc}})$ are 0.567 M and 0.595 M, and 0.713 M, for partial orders (α, β) equal to (0.7,0.7), (0.7,0.6), and (0.5,0.5). The number of degrees of freedom is 58. The comparison over the (0.5,0.5) model gives F-ratios $F = 0.632$ and $F = 0.696$ for the (0.7,0.7) and the (0.7,0.6) alternatives, respectively. As $F(0.05, 58, 58) = 0.646$, the model with non-half-integer orders (0.7,0.7) is statistically significant at the 5% level, while model (0.7,0.6) appears not significant.

- **Fig. S6:** The contour levels of relative errors on the kinetic constant show that differences in the location of the minima occur on changing the dataset. The data needed to assess whether partial orders which are not semi-integers pass the $F-$tests, as compared with the closest semi-integers are shown in Table S12.

Table S12: Statistical data needed to assess whether partial orders which are not semi-integers pass the $F-$tests, as compared with the closest semi-integers.

Sets	Table	$s_{0.5}$ (mM)	$s_{0.1}$ (mM)	$(\alpha;\beta)$	ν	$F_{\mathrm{crit}}(0.05,\nu,\nu)$	$F < F_{\mathrm{crit}}$
1,2	S17	195	84.2	(0.7;0.8)	39	0.587	yes
1,3	S17	780	665	(0.4;0.7)	38	0.583	no
2,3	S17	761	672	(0.7;0.7)	38	0.583	no
1	S17	85.1	12.1	(0.0;0.8)	19	0.461	yes
2	S17	129	18.3	(0.1;1.1)	19	0.461	yes
3	S17	638	638	(1.0;0.6)	18	0.451	no
1,2	S18	82.3	20.4	(0.6;0.6)	41	0.595	yes
1	S18	85.8	18.0	(0.6;0.6)	20	0.471	yes

S6 Numerical data used for the kinetic analysis

In this section, we report the numerical data used for the kinetic analysis discussed in the main text, together with the reference from which the data have been extracted.

Table S13: Time variation of the concentration of **1** according to ref. 21. Data used for Fig. 1 of our work.

t (min)	[A] (M)
0	0.0053
5	0.0049
10	0.0044
15	0.0040
20	0.0036
25	0.0034
30	0.0032
35	0.0030
45	0.0026
55	0.0024
65	0.0021
75	0.0020
120	0.0015

Table S14: Time variation of the concentration of **3** according to ref. 27. Data used for Fig. 2 of our work.

t (min)	[A] (M)
0	0.0500
20	0.0400
40	0.0320
60	0.0260
80	0.0200
100	0.0150
120	0.0100
0	0.0750
30	0.0590
60	0.0430
90	0.0310
120	0.0220
150	0.0130
0	0.1000
30	0.0780
60	0.0620
90	0.0490
120	0.0360
150	0.0240

Table S15: Time variation of concentration of reactants in the A + B $\longrightarrow$ P reaction, retrieved from Tab. IV.2 of ref. 17. Data used for Fig. 3 of our work.

t (s)	[A] (M)	[B] (M)
0	0.0962434	0.04921004
720	0.0857868	0.03875344
1200	0.0804518	0.03341844
1800	0.07486072	0.02782736
2520	0.06986716	0.0228338
3060	0.06709296	0.0200596
3780	0.06384928	0.01681592

Table S16: Time variation of concentration of reactants and product in the A + B $\longrightarrow$ P reaction, for a concentration of catalyst equal to 0.01 M. Data retrieved from Section 6.1 of Supporting Information of ref. 4. Data used for Fig. 4 of our work.

t (h)	[A] (M)	[B] (M)	[P] (M)
0	0.998	0.6	0.001
0.89	0.85	0.449	0.15
2.03	0.726	0.326	0.273
3.89	0.609	0.209	0.388
6.61	0.522	0.123	0.477
10	0.468	0.067	0.534
14.03	0.435	0.036	0.564
18.36	0.417	0.018	0.582
22.97	0.409	0.009	0.592
27.78	0.403	0.004	0.595
0	0.602	0.601	0
0.67	0.525	0.524	0.075
2.67	0.382	0.382	0.218
5.67	0.272	0.272	0.329
12.11	0.164	0.166	0.433
19.11	0.118	0.116	0.483
25.22	0.093	0.093	0.508
46.89	0.055	0.056	0.546
89.22	0.028	0.029	0.573
111.11	0.021	0.025	0.578

Table S17: Time variation of concentration of reactants for the hydrolitic kinetic resolution of epoxides described in ref.[25] These data, retrieved from Section 6.3 of Supporting Information of ref. 4, have been used for Fig. S5 of our work.

t (h)	[**5**] (M)	water (M)
0	6.1900	3.6300
0.0800	5.7710	3.2130
0.1700	5.3960	2.8380
0.2500	5.0540	2.4960
0.3300	4.7450	2.1870
0.4200	4.4670	1.9100
0.5000	4.2180	1.6620
0.5800	3.9970	1.4410
0.6700	3.8020	1.2470
0.7500	3.6310	1.0760
0.8300	3.4810	0.9260
0.9200	3.3510	0.7960
1.0000	3.2380	0.6830
1.0800	3.1400	0.5850
1.1700	3.0550	0.5010
1.2500	2.9820	0.4280
1.3300	2.9200	0.3660
1.4200	2.8660	0.3120
1.5000	2.8200	0.2660
1.5800	2.7810	0.2270
1.6700	2.7470	0.1940
0	4.0000	3.6300
0.1200	3.5160	3.1430
0.2500	3.0980	2.7260
0.3700	2.7360	2.3640
0.4900	2.4250	2.0530
0.6100	2.1580	1.7870
0.8600	1.7370	1.3660
1.1000	1.4310	1.0610
1.3500	1.2070	0.8370
1.5900	1.0410	0.6710
1.8400	0.9160	0.5470
2.0800	0.8200	0.4510
2.3300	0.7450	0.3760
2.5700	0.6860	0.3170
2.8200	0.6380	0.2690
3.0600	0.5990	0.2300
3.3100	0.5670	0.1980
3.5500	0.5400	0.1710
3.8000	0.5180	0.1490
4.0400	0.4990	0.1300
4.2900	0.4830	0.1140
0	6.1900	2.6300
0.1200	5.7070	2.1520
0.2300	5.3020	1.7470
0.3500	4.9600	1.4050
0.4700	4.6750	1.1210
0.5800	4.4410	0.8880
0.7000	4.2510	0.6980
0.8200	4.0990	0.5470
0.9400	3.9780	0.4260
1.0500	3.8830	0.3310
1.1700	3.8080	0.2560
1.2900	3.7490	0.1980
1.4000	3.7040	0.1520
1.5200	3.6690	0.1170
1.6400	3.6410	0.0900
1.7500	3.6210	0.0690
1.8700	3.6040	0.0530
1.9900	3.5920	0.0410
2.1100	3.5820	0.0310
2.2200	3.5750	0.0240

Table S18: Time variation of the concentration of epoxide **5** and water according to the same kinetic parameters used in Section 6.3 of Supporting Information of ref.[4] ($[\text{cat}]_T$= 0.0308 M; $k_{\text{epo}} = 10^6$ M^{-1} s^{-1}; $k_{-\text{epo}} = 8.4890 \cdot 10^6$ s^{-1}; $k_{\text{H}_2\text{O}} = 10^6$ M^{-1} s^{-1}; $k_{-\text{H}_2\text{O}} = 5.5556 \cdot 10^6$ s^{-1}; $k_r = 17.88$ M^{-1} s^{-1}). These data, generated using COPASI 4.30[29] to add a set with a stoichiometric condition ($[\mathbf{5}]_0$=$[H_2O]_0$=4.91 M), have been used for Fig. S5 of our work.

t (s)	[**5**] (M)	water (M)
0	6.19	3.63
288	5.7871	3.22847
576	5.42489	2.86665
864	5.09351	2.53566
1152	4.79219	2.23473
1440	4.51986	1.96279
1728	4.27517	1.71847
2016	4.05652	1.50018
2304	3.86218	1.30619
2592	3.69027	1.1346
2880	3.53886	0.983497
3168	3.40605	0.850962
3456	3.28994	0.735117
3744	3.18876	0.63417
4032	3.10082	0.546439
4320	3.02456	0.470369
4608	2.95857	0.40454
4896	2.90155	0.34767
5184	2.85235	0.298608
5472	2.80996	0.256334
5760	2.77347	0.219947
6048	2.74209	0.188653
0	4.91	4.91
288	4.5057	4.50202
576	4.13647	4.13292
864	3.79462	3.79122
1152	3.47948	3.47621
1440	3.19011	3.18698
1728	2.9254	2.92241
2016	2.68406	2.68121
2304	2.46468	2.46196
2592	2.26574	2.26316
2880	2.0857	2.08324
3168	1.92301	1.92067
3456	1.77613	1.77391
3744	1.6436	1.64149
4032	1.52403	1.52203
4320	1.41612	1.41422
4608	1.31866	1.31686
4896	1.23056	1.22885
5184	1.15083	1.14919
5472	1.07856	1.077
5760	1.01294	1.01146
6048	0.953275	0.951859

S7 Manual of IPLOT-VKA Web-Application

This section describes the use of our Web-Application, which allows the use of Powell-Margerison Analysis to retrieve retrieve total and partial orders of reaction for an A + B $\rightarrow$ P reaction as explained in the main text.

The manual is organized so that each section title correspond to one of the features available in the site itself.

An updated version of this manual can be found at this link

S7.1 Examples

Together with the tutorial, the website provides the four examples discussed in the main text. By checking one of the boxes the corresponding kinetic data will be analysed. Notice that the numerical data used for each example are shown by simply passing the mouse pointer over the corresponding nametag The Output Page shown after the calculation reports a reference to the scientific paper from which the data have been retrieved

S7.2 New analysis

There are two different parts: Mandatory Input Data (essentially, a txt file with the Kinetic Data) and Optional Input Data. Each Input part is described in the following sections:

Kinetic data

Upload a ".txt" file with either 2 or 3 columns. The columns can be separated by spaces, indentations or commas. *WARNING*: Only files with ".txt" extension are accepted 2-column file must contain time as first column; concentration as second column. 3-column file must contain time as first column; concentration of the two reagents as second and third column, respectively.

Total Order Analysis

Indicate the minimum, the maximum and the step for the range of orders to be explored.

This grid will be used for the exploration of the total order, unless the latter is explicitly indicated by the user (see below). In this latter case, the grid will be used for the exploration of the partial orders BE CAREFUL: if you set incompatible values, this may lead to errors: if unsure, consider leaving these boxes empty! Put in the first box the smallest order; in the second box the highest order; in the third box the step. For example, indicating "-1" in the first box; "3" in the second and "0.5" in the third, leads to the default analysis of the orders (-1.0, -0.5, 0.0, 0.5, 1.0, 1.5, 2.0, 2.5, 3.0) *WARNING*: If wrong or no values are given, only these default orders will be explored

Partial Order Analysis

If you want to investigate partial orders only, you can indicate the total reaction order in the box "Total reaction order:" (Default = no order given)

If you want to perform a systematic search over partial orders of reactions, check the box "Systematic search over partial orders" *WARNING*: If you set a too dense grid, the "Systematic search" can fail! This is due to the fact that the web-app has been set to give a quick response, therefore the site has a computational time limit. In that case, consider analyzing a smaller number of partial orders at first and then refining them. For example, if you want to analyze all the possible combinations of partial orders between -1 and +3 in step of 0.1, this would lead to a 1681-points grid!

Analytic or Approximate (default) search?

If you want to resort to analytic (hypergeometric) analysis, check the box "Use analytic method". Otherwise, the approximated solution f_{IV} will be used. The approximate solution f_{IV} matches the hypergeometric solution exactly for 79 out of the 81 points in the 0.5-unit spaced grid of the [-1,3] $\times$ [-1,3] domain of α and β orders, as discussed above.

S7.3 Data produced

In output, the user will be redirected to a page where the Powell plot are shown together with the best (total and/or partial) order predicted and the corresponding errors.

The Output Page is divided in two sections.

The first section reports the Main Results (i.e. kinetic constants and total/partial orders of reactions) which represents the information that the majority of the users are interested in. Here we also provide the residual standard error on concentration, which measures how well the simple kinetic model describe the experimental data and it is calculated as in Eq. S24

WARNING: please, consider whether the reduction of s with the finer grid is statistically significant, as discussed in Section S5.

The second section ("Further Analysis") reports a more-in-depth analysis, reporting a point-to-point description of the kinetic constants and errors predicted in the Powell-Margerison Analysis, together with the corresponding plots. This second part is deemed necessary for the users interested in a more detailed study of the prediction performed by the methodology. All the plots can be saved as images. In this section we also provide a link to download the master curves (either ϕ vs $\log_{10}(t/t^*)$ or Δf vs $t/t*$) as csv files for possible further analysis to be performed by the user outside the Web-App

References

(1) Blackmond, D. G. Reaction Progress Kinetic Analysis: A Powerful Methodology for Mechanistic Studies of Complex Catalytic Reactions. *Angewandte Chemie International Edition* **2005**, *44*, 4302–4320.

(2) Baxter, R. D.; Sale, D.; Engle, K. M.; Yu, J.-Q.; Blackmond, D. G. Mechanistic Rationalization of Unusual Kinetics in Pd-Catalyzed C–H Olefination. *Journal of the American Chemical Society* **2012**, *134*, 4600–4606.

(3) Burés, J. A Simple Graphical Method to Determine the Order in Catalyst. *Angewandte Chemie* **2016**, *128*, 2068–2071.

(4) Burés, J. Variable Time Normalization Analysis: General Graphical Elucidation of Reaction Orders from Concentration Profiles. *Angewandte Chemie International Edition* **2016**, *55*, 16084–16087.

(5) Nielsen, C. D.-T.; Burés, J. Visual kinetic analysis. *Chemical Science* **2019**, *10*, 348–353.

(6) Benson, S. W. *Foundations of Chemical Kinetics*; Mc Graw Hill: New York, 1960; [Online; accessed 2023-06-19].

(7) Frost, A. A.; Pearson, R. G. *Kinetics and mechanism: a study of homogeneous chemical reactions*, 2nd ed.; Wiley: New York, 1965; OCLC: 421443412.

(8) Levine, I. N. *Physical chemistry*, 5th ed.; McGraw-Hill: Boston, 2002.

(9) Espenson, J. H. *Chemical kinetics and reaction mechanisms*, 2nd ed.; McGraw-Hill Custom Publishing: New York [etc.], 2007; OCLC: 803452475.

(10) Margerison, D. In *The practice of kinetics*; Bamford, C. H., Tipper, C. F. H., Eds.; Comprehensive chemical kinetics. Section 1: The practice and theory of kinetics, v. 1; Elsevier: Amsterdam, New York [etc.], 1969.

(11) Tanemura, K. A.; Sierra-Costa, D.; Merz, K. M. *Python for Chemists*; ACS In Focus; American Chemical Society: Washington, DC, USA, 2022; DOI: 10.1021/acsinfocus.7e5030.

(12) Mills, I. In *Quantities, units, and symbols in physical chemistry*, 3rd ed.; of Pure, I. U., Chemistry, A., Eds.; RSC Pub: Cambridge, UK, 2007.

(13) Perrin, C. L. et al. Glossary of terms used in physical organic chemistry (IUPAC Recommendations 2021). *Pure and Applied Chemistry* **2022**, *94*, 353–534.

(14) Muller, P. Glossary of terms used in physical organic chemistry (IUPAC Recommendations 1994). *Pure and Applied Chemistry* **1994**, *66*, 1077–1184.

(15) Kacser, H.; Burns, J. A.; Kacser, H.; Fell, D. A. The control of flux. *Biochemical Society Transactions* **1995**, *23*, 341–366.

(16) Burés, J. What is the Order of a Reaction? *Topics in Catalysis* **2017**, *60*, 631–633.

(17) Benson, S. W. *Foundations of Chemical Kinetics*; Mc Graw Hill: New York, 1960; pp 75–77, [Online; accessed 2023-06-19].

(18) Roseveare, W. E. Methods Of Calculating And Averaging Rate Constants. *Journal of the American Chemical Society* **1931**, *53*, 1651–1661.

(19) Farebrother, R. W. Relations Among Subset Estimators: A Bibliographical Note. *Technometrics* **1985**, *27*, 85–86, 00013.

(20) Monaco, G.; Fedullo, A. Teaching Least Squares in Matrix Notation. *Ratio Mathematica - Rivista di Matematica, Statistica ed Applicazioni* **2017**, 00000.

(21) Weiss, H. M.; Touchette, K. A simple second-order kinetics experiment. *Journal of Chemical Education* **1990**, *67*, 707.

(22) Hancock, L. M.; McGarvey, D. J.; Plana, D. An Investigation of the Temperature Dependence of a Monomer–Dimer Equilibrium Using UV–Vis and ^{1} H NMR Spectroscopies. *Journal of Chemical Education* **2023**, *100*, 1283–1288.

(23) Wang, Y.; Liao, W.; Wang, Y.; Jiao, L.; Yu, Z.-X. Mechanism and Stereochemistry of Rhodium-Catalyzed [5 + 2 + 1] Cycloaddition of Ene–Vinylcyclopropanes and Carbon Monoxide Revealed by Visual Kinetic Analysis and Quantum Chemical Calculations. *Journal of the American Chemical Society* **2022**, *144*, 2624–2636.

(24) Weisstein, E. W. Hypergeometric Function. `https://mathworld.wolfram.com/HypergeometricFunction.html`, publisher: Wolfram Research, Inc.

(25) Ford, D. D.; Nielsen, L. P. C.; Zuend, S. J.; Musgrave, C. B.; Jacobsen, E. N. Mechanistic Basis for High Stereoselectivity and Broad Substrate Scope in the (salen)Co(III)-Catalyzed Hydrolytic Kinetic Resolution. *Journal of the American Chemical Society* **2013**, *135*, 15595–15608.

(26) Press, W. H., Ed. *FORTRAN Numerical Recipes*, 2nd ed.; Cambridge University Press: Cambridge [England] ; New York, 1996.

(27) Wang, Y.; Liao, W.; Wang, Y.; Jiao, L.; Yu, Z.-X. Mechanism and Stereochemistry of Rhodium-Catalyzed [5 + 2 + 1] Cycloaddition of Ene–Vinylcyclopropanes and Carbon Monoxide Revealed by Visual Kinetic Analysis and Quantum Chemical Calculations. *J. Am. Chem. Soc.* **2022**, *144*, 2624–2636.

(28) Nielsen, L. P. C.; Stevenson, C. P.; Blackmond, D. G.; Jacobsen, E. N. Mechanistic Investigation Leads to a Synthetic Improvement in the Hydrolytic Kinetic Resolution of Terminal Epoxides. *J. Am. Chem. Soc.* **2004**, *126*, 1360–1362.

(29) Hoops, S.; Sahle, S.; Gauges, R.; Lee, C.; Pahle, J.; Simus, N.; Singhal, M.; Xu, L.; Mendes, P.; Kummer, U. COPASI- A COmplex PAthway SImulator. *Bioinformatics* **2006**, *22*, 3067–3074.
